# Ability of ChatGPT to Replace Doctors in Patient Education: Cross-Sectional Comparative Analysis of Inflammatory Bowel Disease

**DOI:** 10.2196/62857

**Published:** 2025-03-31

**Authors:** Zelin Yan, Jingwen Liu, Yihong Fan, Shiyuan Lu, Dingting Xu, Yun Yang, Honggang Wang, Jie Mao, Hou-Chiang Tseng, Tao-Hsing Chang, Yan Chen

**Affiliations:** 1 Zhejiang Provincial Key Laboratory of Gastrointestinal Diseases Pathophysiology Department of Gastroenterology The First Affiliated Hospital of Zhejiang Chinese Medical University Hangzhou China; 2 Center of Inflammatory Bowel Diseases Department of Gastroenterology The Second Affiliated Hospital, Zhejiang University School of Medicine Hangzhou China; 3 The China Crohn’s & Colitis Foundation Hangzhou China; 4 The Clinical Medical College Zhejiang University School of Medicine Hangzhou China; 5 Department of Gastroenterology The Affiliated Huaian No.1 People’s Hospital of Nanjing Medical University Huai'an China; 6 The Second Clinical Medical College Zhejiang Chinese Medical University Hangzhou China; 7 Graduate Institute of Digital Learning and Education National Taiwan University of Science and Technology Taipei Taiwan; 8 Department of Computer Science and Information Engineering National Kaohsiung University of Science and Technology Kaohsiung Taiwan

**Keywords:** AI-assisted, patient education, inflammatory bowel disease, artificial intelligence, ChatGPT, patient communities, social media, disease management, readability, online health information, conversational agents

## Abstract

**Background:**

Although large language models (LLMs) such as ChatGPT show promise for providing specialized information, their quality requires further evaluation. This is especially true considering that these models are trained on internet text and the quality of health-related information available online varies widely.

**Objective:**

The aim of this study was to evaluate the performance of ChatGPT in the context of patient education for individuals with chronic diseases, comparing it with that of industry experts to elucidate its strengths and limitations.

**Methods:**

This evaluation was conducted in September 2023 by analyzing the responses of ChatGPT and specialist doctors to questions posed by patients with inflammatory bowel disease (IBD). We compared their performance in terms of subjective accuracy, empathy, completeness, and overall quality, as well as readability to support objective analysis.

**Results:**

In a series of 1578 binary choice assessments, ChatGPT was preferred in 48.4% (95% CI 45.9%-50.9%) of instances. There were 12 instances where ChatGPT’s responses were unanimously preferred by all evaluators, compared with 17 instances for specialist doctors. In terms of overall quality, there was no significant difference between the responses of ChatGPT (3.98, 95% CI 3.93-4.02) and those of specialist doctors (3.95, 95% CI 3.90-4.00; *t*_524_=0.95, *P*=.34), both being considered “good.” Although differences in accuracy (*t*_521_=0.48, *P*=.63) and empathy (*t*_511_=2.19, *P*=.03) lacked statistical significance, the completeness of textual output (*t*_509_=9.27, *P*<.001) was a distinct advantage of the LLM (ChatGPT). In the sections of the questionnaire where patients and doctors responded together (Q223-Q242), ChatGPT demonstrated inferior performance (*t*_36_=2.91, *P*=.006). Regarding readability, no statistical difference was found between the responses of specialist doctors (median: 7th grade; Q1: 4th grade; Q3: 8th grade) and those of ChatGPT (median: 7th grade; Q1: 7th grade; Q3: 8th grade) according to the Mann-Whitney U test (*P*=.09). The overall quality of ChatGPT’s output exhibited strong correlations with other subdimensions (with empathy: *r*=0.842; with accuracy: *r*=0.839; with completeness: *r*=0.795), and there was also a high correlation between the subdimensions of accuracy and completeness (*r*=0.762).

**Conclusions:**

ChatGPT demonstrated more stable performance across various dimensions. Its output of health information content is more structurally sound, addressing the issue of variability in the information from individual specialist doctors. ChatGPT’s performance highlights its potential as an auxiliary tool for health information, despite limitations such as artificial intelligence hallucinations. It is recommended that patients be involved in the creation and evaluation of health information to enhance the quality and relevance of the information.

## Introduction

In the medical field, large language models (LLMs), represented by ChatGPT, have shown significant application potential: In oncology, various brands of LLMs consistently generate relatively accurate and high-quality information, highlighting their potential as sources of medical information [1]. From English to Chinese environments, LLMs have impressively passed their respective medical licensing exams, demonstrating their communication abilities in multilingual clinical settings and their foundational potential in medical education [2-4]. Whether the input text is everyday medical records or structured terminological reports, ChatGPT can swiftly interpret cues based on evidence-based guidelines, aiding health care providers in making informed decisions and showing significant potential in enhancing patient follow-up adherence [5,6]. LLMs have showcased their “rich medical knowledge” and the ability to extract disease information from various languages and contexts. Their method of providing information in a “human-like” tone is considered more effective than traditional search engines [7]. Despite a lack of evidence, these tools are being adopted by patients and clinical doctors [6,8]. The reason behind their excellent performance is that their text training set comes from a vast amount of publicly available internet information, making the quality of medical information provided by LLMs comparable to existing internet information [9,10].

Patients with chronic diseases themselves exhibit higher enthusiasm and realistic motivation in seeking health information and using web-based health technologies [11]. Undoubtedly, as artificial intelligence (AI) applications become more widespread, an increasing number of patients will use this technology in practice, and the quality of health information can have a positive or negative impact on patients’ clinical outcomes [12,13]. In the field of inflammatory bowel disease (IBD), the evaluation reports of health information, whether in Chinese or English, have been consistently mediocre, once deemed insufficient to meet patient needs [14-18]. When emerging AI tools replace static traditional internet information, the impact on patient education and self-management requires careful consideration and evaluation.

Including Crohn disease and ulcerative colitis, IBD is an increasingly prevalent chronic intestinal disease in China, characterized by primary invasion of the digestive system and cumulative multisystem involvement of autoimmune diseases, with no cure currently available. Patients have a strong need to learn and reinforce self-care abilities, among which the WeChat public account of the China Crohn’s and Colitis Foundation (CCCF) is the most popular with IBD patients [19]. We used it as a representative to study the patient education ecosystem for chronic diseases.

This study aimed to evaluate ChatGPT’s ability to provide specialized vertical domain information, especially in the education of some patients with chronic disease, and compare it with industry experts. Through this comparison, we can identify the strengths and limitations of LLMs in medical information services, providing a basis for further improvement and application of the technology. Additionally, this study aimed to enhance the public and medical professionals’ awareness and acceptance of using AI tools in medical information acquisition and education.

## Methods

### Collection of Questions and the Original Doctor Responses

The mode of one-on-one question-and-answer dialogue stands as a prevalent form of interaction within the health care domain. Across various medical applications, online forums, and instant messaging groups, a substantial portion of queries manifest as repetitive and amenable to categorization.

In earlier epochs, we undertook the aggregation of high-frequency, prototypical questions posed by patients with IBD through online platforms and outpatient settings. We extended invitations to industry peers to collectively address these patient queries, culminating in the publication of a didactic tome titled “Q&A on Ulcerative Colitis and Crohn’s Disease” tailored for the self-learning of individuals with inflammatory bowel ailments. This publication reflects the cumulative outcomes of doctor-patient interactions over 7 years at the CCCF and the Second Affiliated Hospital-Zhejiang University School of Medicine IBD Center encompassing 9000 cases. The compendium was predominantly curated by 8 seasoned IBD specialist doctors, with contributions from 55 IBD practitioners and 5 experienced patients with a high level of cultural acumen. Upon its publication, the book garnered commendations and accolades from numerous esteemed figures within the IBD community in China and the United States. The content delves into various aspects of IBD, including etiology, symptoms, diagnosis, treatment, follow-up protocols, and emotional support. The questions encapsulated within are highly representative and encompass a broad spectrum (Multimedia Appendix 1). Many analogous questions have surfaced on pertinent social media platforms, with the content of this tome serving as a primary source of representative patient inquiries. Presently, the book has undergone 9 printings, with a distribution nearing 20,000 copies.

**Figure 1:**
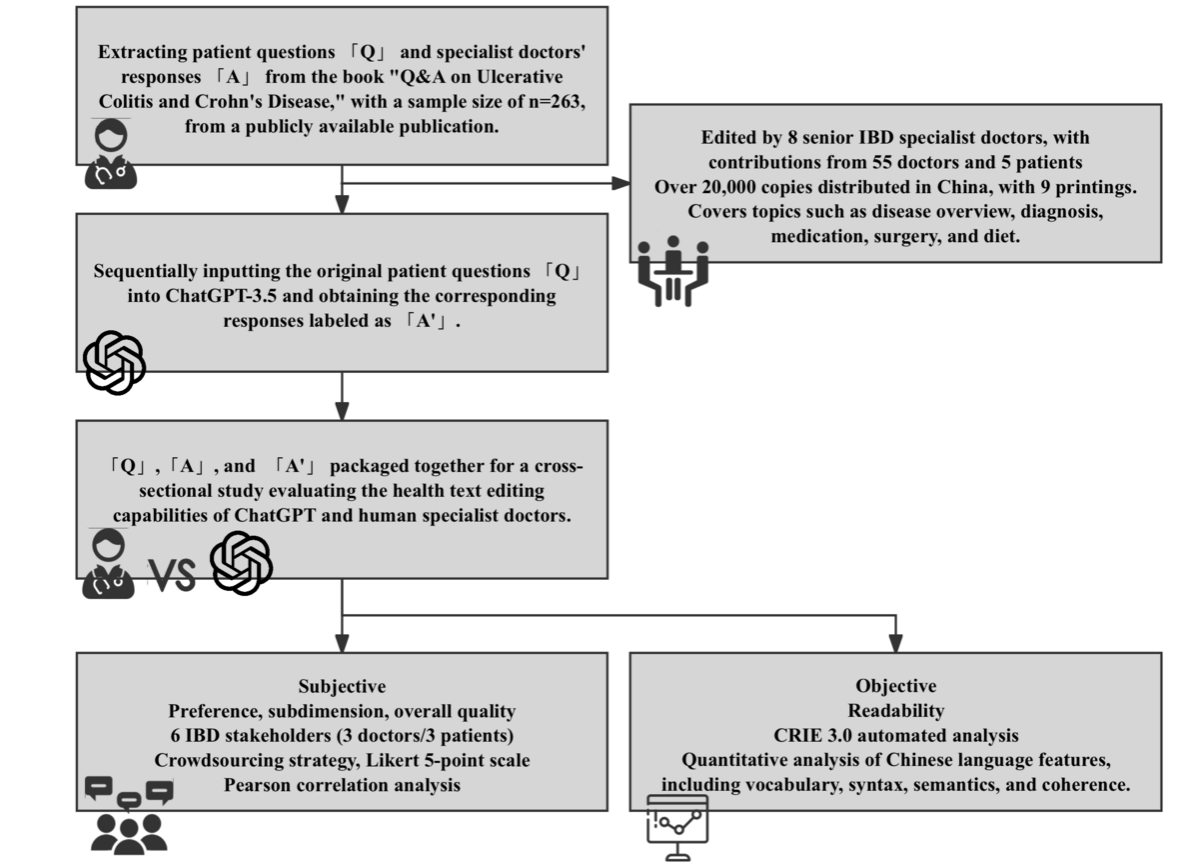
Schematic of operational workflow. CRIE: Chinese Readability Index Explorer; IBD: inflammatory bowel disease.

The thematic essence of the book comprises 263 distinct questions matched with corresponding responses from doctors. Apart from a minor subset of emotional support content provided by patients, all responses are underpinned by evidence-based rationale. This sample size is anticipated to afford us a statistical power of 90% to discern a 10% differential between responses generated by ChatGPT and those proffered by medical practitioners (55% vs 45%; Figure 1).

To enhance the transparency and reproducibility of the experimental results, we did not use anonymized online medical consultation text for doctor-patient interactions. The content in the book was derived from authentic doctor-patient interactions, pre-authorized and publicly disclosed, with many excerpts being republished on social media platforms in electronic format [20]. The data used in this study are publicly available and do not contain any identifiable personal information.

### Ethical Considerations

Based on the assessment by the ethics committee, considering the nature and purpose of the research materials, as well as the practices in prior similar studies, it was determined that this study did not involve direct research on human participants and ultimately did not require ethical review [1,7,9,21-23]. The content is used under authorization and license from Zhejiang University Press.

### Collecting ChatGPT Responses

In the period from September 8, 2023, to September 22, 2023, ChatGPT responses were collected by inputting the original question text into a new chatbot session (GPT-3.5 version, OpenAI, August 3 version, 2023) and saving the chatbot replies [24]. Differing from some other experimental methodologies, we adopted a sequential prompting of all questions listed in the directory within the same bot link [4,23]. The rationale behind this approach includes the following.

First, the original questions in the book contained terminology descriptions presumed to be familiar and comprehensible to health care professionals; for instance, in Chinese, the term [激素] “hormone” in the book and in IBD doctor-patient communication scenarios often specifically refers to [糖皮质激素] “glucocorticoids.” In a typical context, using [激素] “hormone” in communication may commonly lead individuals to think of “chemical messengers between cells,” and according to LLM principles, ChatGPT would respond to the latter in the absence of contextual elucidation. This measure was taken to mitigate the potential for the bot to provide accurate “incorrect responses” due to a lack of contextual background.

Second, upon encountering the instances of the bot misinterpreting the language context, we continued to supplement vocabulary prompts to guide ChatGPT in understanding the true intent of the questioner, thereby eliciting a response that aligned with it. However, prompts were limited to no more than 3 times, drawing from the routine search habits of patients on the web and previous experiments [4,25].

Third, to emulate the habitual reading practices and context of normal situations, we posed questions to ChatGPT in the same sequential order as presented in the book.

### Quality Control

First, the final analytic sample encompassed 263 questions and their corresponding responses from doctors and ChatGPT, as featured in the ninth edition of “Q&A on Ulcerative Colitis and Crohn’s Disease” printed in April 2022. Responses from doctors were designated as the benchmark.

Second, some original responses in the book were provided by patients, and we retained this text, as it had undergone professional medical review before the book’s publication. It can be understood that, although drafted by patient volunteers, the expressions were approved by doctors and deemed suitable for new patients to view, primarily addressing psychological issues (Multimedia Appendix 2).

Third, original illustrations from the book were not excerpted, whereas tables were permitted. This decision was made because, when using ChatGPT, the model itself could generate tables, thus remaining unaffected.

Fourth, at that time, the version of ChatGPT would randomly present 2 response options for user selection when prompted, with the first option being the default choice.

Fifth, due to network issues, in the event of a crash or incomplete display, we would click “regenerate” once to select a complete text answer for material completion.

### Text Content Evaluation 

#### Subjective Assessment 

The assessment was conducted by 6 evaluators (3 licensed IBD doctors and 3 IBD patients). The doctors were experienced IBD physicians in patient education (YC, DX, HW), with over 10 years of clinical experience, having treated more than 500 patients with IBD, and engaged in patient education for over 5 years. The patient characteristics required were individuals aged between 20 years and 60 years, with at least an undergraduate education level, diagnosed with IBD for more than a year, and who had not read the “Questions and Answers” book. To ensure evaluators were as unable as possible to distinguish the source of the text, we used a blind method when presenting the materials to evaluators, concealing explanatory language such as “as an artificial intelligence.” The doctors’ responses and ChatGPT responses for the same question were anonymized and randomly labeled as Response 1 and Response 2. Evaluators were required to first read the question along with the corresponding doctors’ responses and ChatGPT response, followed by a 2-step evaluation process: (1) selecting the preferred answer version and (2) subjectively rating the 2 answers on a 5-point Likert scale for overall quality and dimensional evaluation, referencing dimensions from previous health information research [15,16,23,25], including accuracy, empathy, and completeness. A higher score indicates greater evaluator approval of the response text’s performance in that dimension (see Table 1 for details).

**Table 1:** Definitions of each dimension and pretraining required for evaluators.

Dimension	Definition
Accuracy	Whether the response scientifically and impartially explains the issue, such as providing explanations on medication use and dosage, and clarifies surgical timing limitations
Completeness	Whether there are any omissions of important information or concepts in the explanation
Empathy	Whether the response demonstrates an understanding of the question from the perspective of the “patient” or the inquirer
Overall quality	Subjective perception of the overall quality of the text

#### Objective Evaluation

The Chinese Readability Index Explorer (CRIE; version 3.0 [26]) was used. In addition to the evaluators’ subjective assessments, we introduced a quantitative Chinese readability tool, CRIE. It consists of 4 subsystems comprising 82 multilevel language features [27]. CRIE uses multilevel language features for text analysis, including vocabulary, syntax, semantics, and cohesion. This tool aids with analyzing various types of texts, such as Chinese textbooks [28], foreign language learning materials [29], and domain-specific knowledge texts [30]. Numerous studies have validated its reliability and practicality in the Chinese health domain [31,32]. Results can be interpreted using the Flesch-Kincaid English readability assessment method: the higher the grade, the greater the text complexity. Quantitative natural language processing and text mining tools serve as valuable supplements to subjective human evaluations [4].

### Data Statistics and Analysis

#### Data Aggregation

Aligned with the research objectives, we used a crowdsourced scoring strategy for data collection, a method that aggregates data across a collective of evaluators. Primarily applied in the field of linguistics, where language use is a fundamental domain for the general populace, the central idea is to harness the collective expertise of both experts and the public to pioneer new concepts through crowd annotations. This method is well-suited for subjective evaluations, such as scoring of singing by judges or the exploration of novel concepts. Calculating average scores for each dimension reflects the consistency variances among evaluators, encapsulating individual uncertainties and subjective biases within the variance of the scores [21]. In the context of health text evaluation, the involvement of judges and the assessment method, involving direct quantification by both IBD health care providers and consumers, represents a feasible, efficient, cost-effective, and relatively accessible evaluation strategy.

#### Primary Outcomes

We conducted descriptive analysis and assessed evaluators’ preference ratios for ChatGPT using a chi-square goodness-of-fit test. A 2-tailed Welch *t* test was used to compare the mean values of the 2 responses. We defined a threshold score of 3 (acceptable) and calculated the proportion exceeding or falling below this threshold score (3), comparing them using prevalence ratios. Furthermore, we evaluated the Pearson correlation coefficients between the various subdimensions of quality to observe or predict correlations between different dimensions. Given that the readability of each response text is a calculated ordinal variable, nonparametric tests were used for comparison.

#### Secondary Outcomes

Subgroup *t* test analyses were conducted to assess the impact of evaluator identity (physician/patient) and the original response creator’s source (solely doctor/doctor-patient collaboration) on mean scores.

A significance level of *P*<.05 was set, and Bonferroni correction was applied for multiple tests. All statistical analyses were performed using R software (version 4.3.1 GUI 1.79 Big Sur ARM build) and RStudio (version 2023.09.1+494). Data visualization was created based on code references from the open-source platform Hiplot.

## Results

### Preferred Response Ratio

Of 1578 evaluations, evaluators showed a preference for ChatGPT responses at a rate of 48.4% (95% CI 45.9%-50.9%; *P*=.20). Among these, 6 evaluators exclusively favored ChatGPT responses for a total of 12 questions: 22, 28, 42, 56, 73, 120, 121, 124, 127, 161, 174, 195. Evaluators exclusively favored doctors’ responses for 17 questions: 5, 27, 41, 83, 85, 92, 156, 175, 180, 198, 205, 210, 219, 234, 237, 251, 263. The questions and corresponding responses from both doctors and the AI model are detailed in Multimedia Appendix 3.

### Comparison of Mean Scores and Prevalence Ratio of Threshold Scores

Overall, the proportion of responses rated below an acceptable quality (<3) was 1.26 times higher for doctors’ responses than for ChatGPT responses (doctors: 3.3%, 95% CI 2.5%-4.4%; ChatGPT: 2.7%, 95% CI 1.9%-3.5%). Simultaneously, the proportion of responses rated as good or very good quality was 1.10 times higher for ChatGPT than for doctors (doctors: 69.4%, 95% CI 67.1%-71.7%; ChatGPT: 76%, 95% CI 73.7%-78%). Although ChatGPT had a slight advantage in the overall quality distribution, there was no significant difference between ChatGPT and doctors’ responses (t_524_=0.95, *P*=.34), with doctors’ (3.95, 95% CI 3.90-4.00) and ChatGPT responses (3.98, 95% CI 3.93-4.02) both rated at a “good” level.

In terms of the completeness dimension, ChatGPT responses significantly outperformed doctors’ responses (t_509_=9.27, *P*<.001), although both doctors’ responses (3.88, 95% CI 3.83-3.94) and ChatGPT responses (4.21, 95% CI 4.17-4.26) were rated at a “good” level. The proportion of low completeness responses was 3.66 times higher for doctors’ responses (6.7%, 95% 5.53%-8.97%) than for ChatGPT responses (1.8%, 95% CI 1.2%-2.6%); the proportion of high completeness responses was 1.20 times higher for ChatGPT responses (81.1%, 95% CI 79.2%-83.1%) than for doctors’ responses (67.4%, 95% CI 65.1%-69.7%). Pretrained models and structured outputs contributed to ChatGPT receiving more favor in this dimension.

In the empathy dimension, ChatGPT responses were inferior to doctors’ responses (t_511_=2.19, *P*=.03). Due to the significance correction for multiple tests, we conservatively state that there is no significant difference between doctors’ responses (3.99, 95% CI 3.95-4.03) and ChatGPT responses (4.06, 95% 4.01-4.11). The proportion of low empathy responses was 1.12 times higher for ChatGPT responses (2.8%, 95% CI 2%-3.7%) than for doctors’ responses (2.5%, 95% 1.8%-3.4%), while the proportion of high empathy responses was 1.04 times higher for ChatGPT responses (75%, 95% CI 72.8%-77.1%) than for doctors’ responses (72%, 95% CI 69.6%-74.1%).

In the accuracy dimension, there was not a significant difference between ChatGPT and doctors’ responses (t_521_=0.48, *P*=.63). Doctors’ responses (4.11, 95% CI 4.07-4.15) and ChatGPT responses (4.12, 95% CI 4.08-4.17) were comparable. The proportion of low accuracy responses was 2.2 times higher for ChatGPT responses (2.4%, 95% CI 1.7%-3.3%) than for doctors’ responses (1.1%, 95% CI 0.6%-1.7%), while the proportion of high-accuracy responses was 1.05 times higher for ChatGPT responses (81.1%, 95% CI 79%-83%) than for doctors’ responses (76.9%, 95% CI 74.7%-78.9%).

### Subgroup Comparisons

#### Discrepancies in Evaluator Perspectives

**Figure 2:**
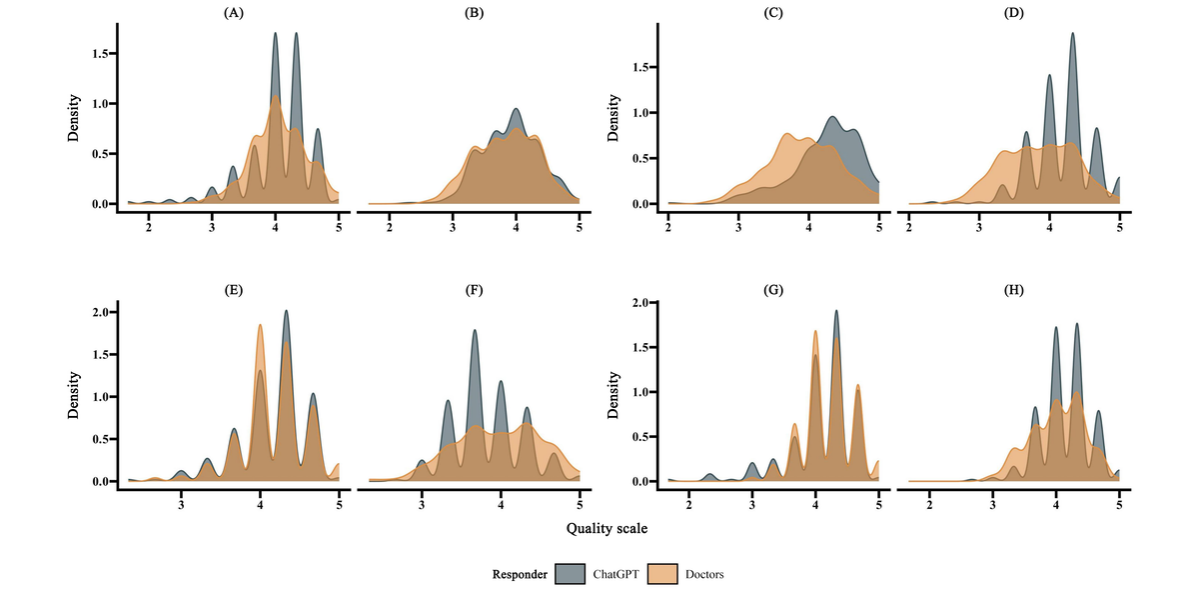
Kernel density plot illustrating the overall quality assessment by (A) physicians and (B) patients, completeness assessment by (C) physicians and (D) patients, empathy assessment by (E) physicians and (F) patients, and accuracy assessment by (G) physicians and (H) patients.

In terms of overall quality, physicians (*P*=.09) and patients (*P*=.88) perceived no difference between ChatGPT and doctors (Figure 2). Regarding completeness, physicians (t_524_=7.7, *P*<.001) and patients (t_508_=8.0, *P*<.001) unanimously agreed that ChatGPT outperformed doctors. On the empathy dimension, although physicians (t_523_=0.38, *P*=.70) did not perceive a difference between the two, patients believed that doctors’ responses exhibited more emotional depth than ChatGPT (t_503_=2.9, *P*=.003). In terms of accuracy, physicians (t_496_=2.3, *P*=.02) considered doctors’ responses to be more accurate, while patients believed that ChatGPT responses held a slight edge in accuracy (t_520_=3.3, *P*<.001).

#### Differences in Responders' Performance

**Figure 3:**
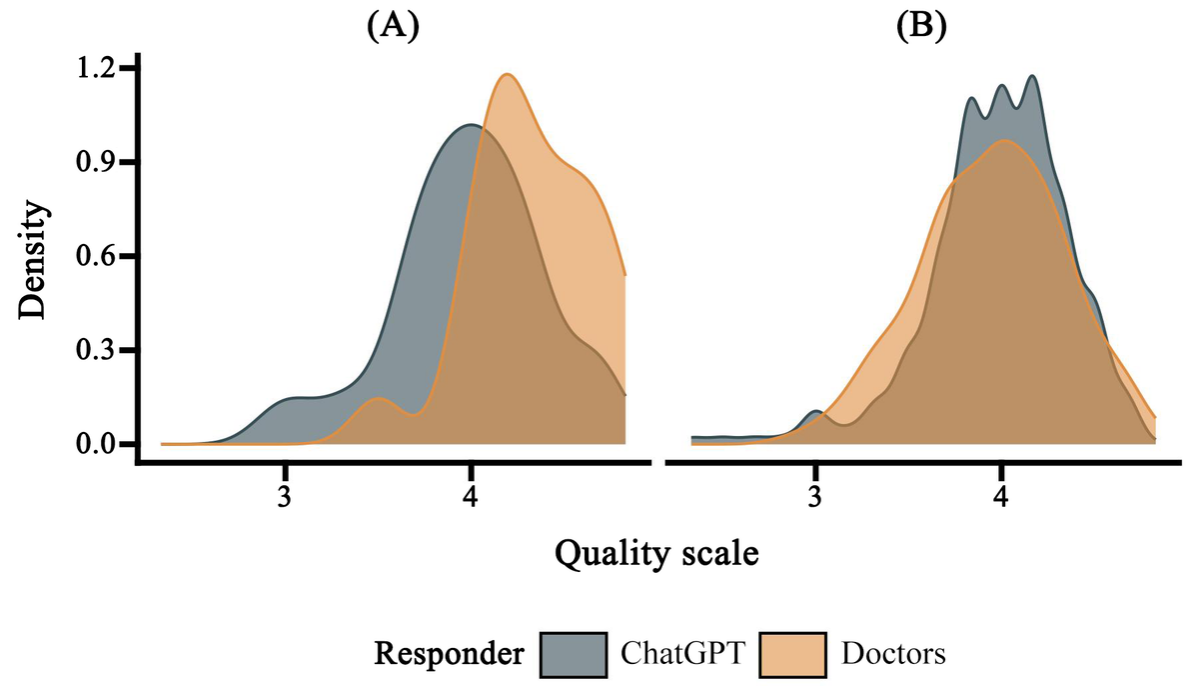
Kernel density plot of the overall quality of the (A) original questions (Q223-Q242) for which patients assisted doctors in crafting responses and (B) all responses.

**Figure 4:**
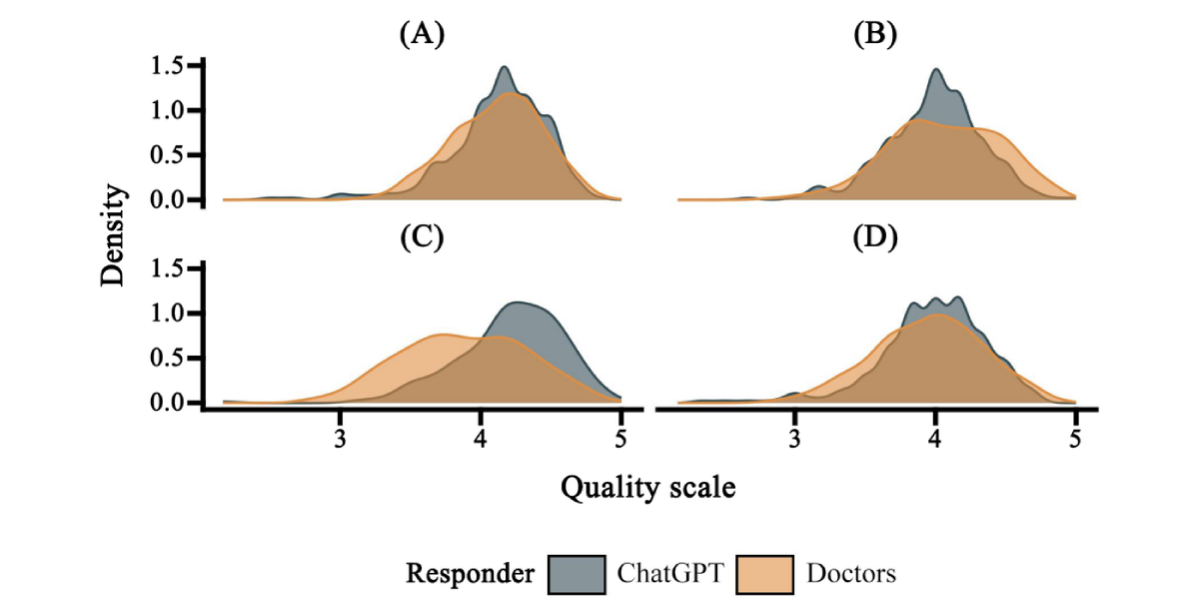
Kernel density plots showing assessors’ evaluations of (A) accuracy, (B) empathy, (C) completeness, and (D) overall quality.

We selected original questions (Q223-Q242) for which patients assisted doctors in crafting responses and found that, in this subset, doctors’ performance significantly surpassed that of ChatGPT (*t*_df_=2.9, *P*=.006; Figure 3). In terms of overall quality and across various dimensions (Figure 4), ChatGPT’s ability to curate health information in the specialized field of medicine is now on par with professional doctors, reaching a level of excellence.

### Subdimension Correlation Analysis

**Figure 5:**
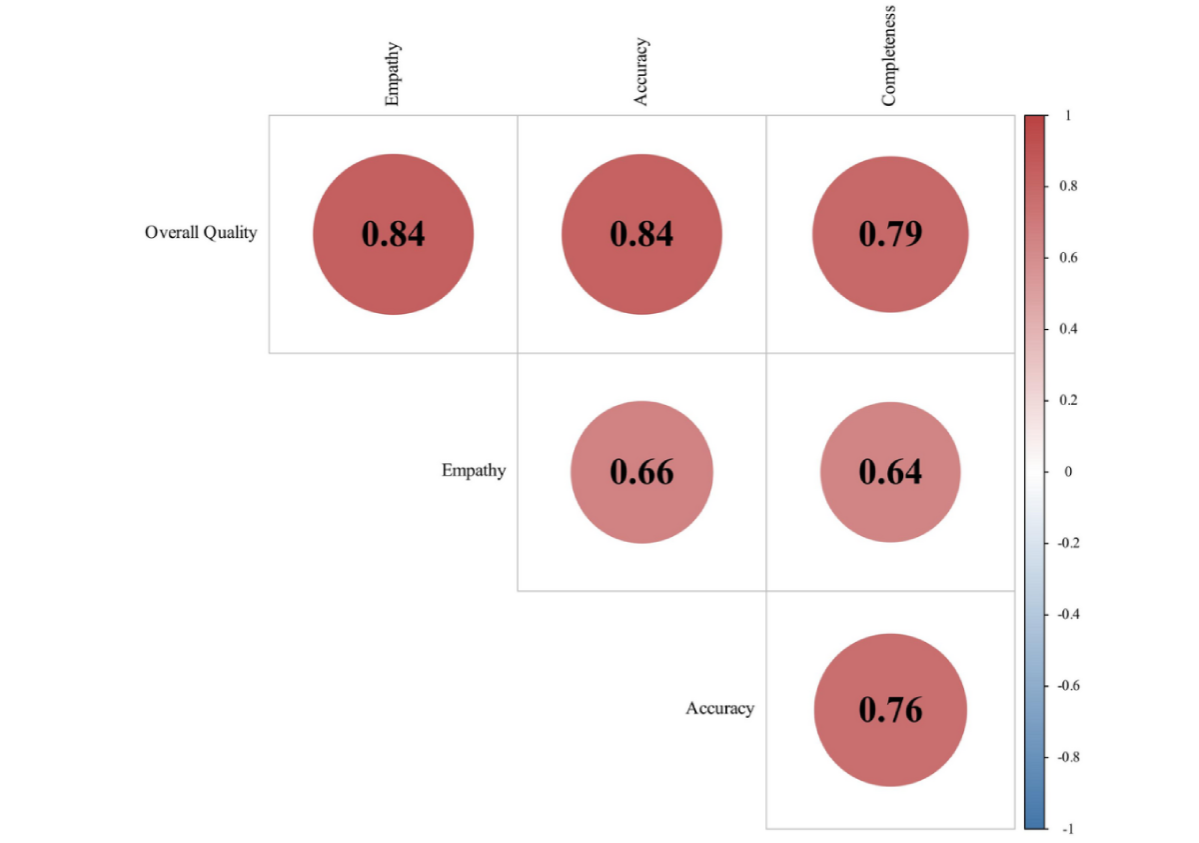
Correlation analysis of ChatGPT responses.

**Figure 6:**
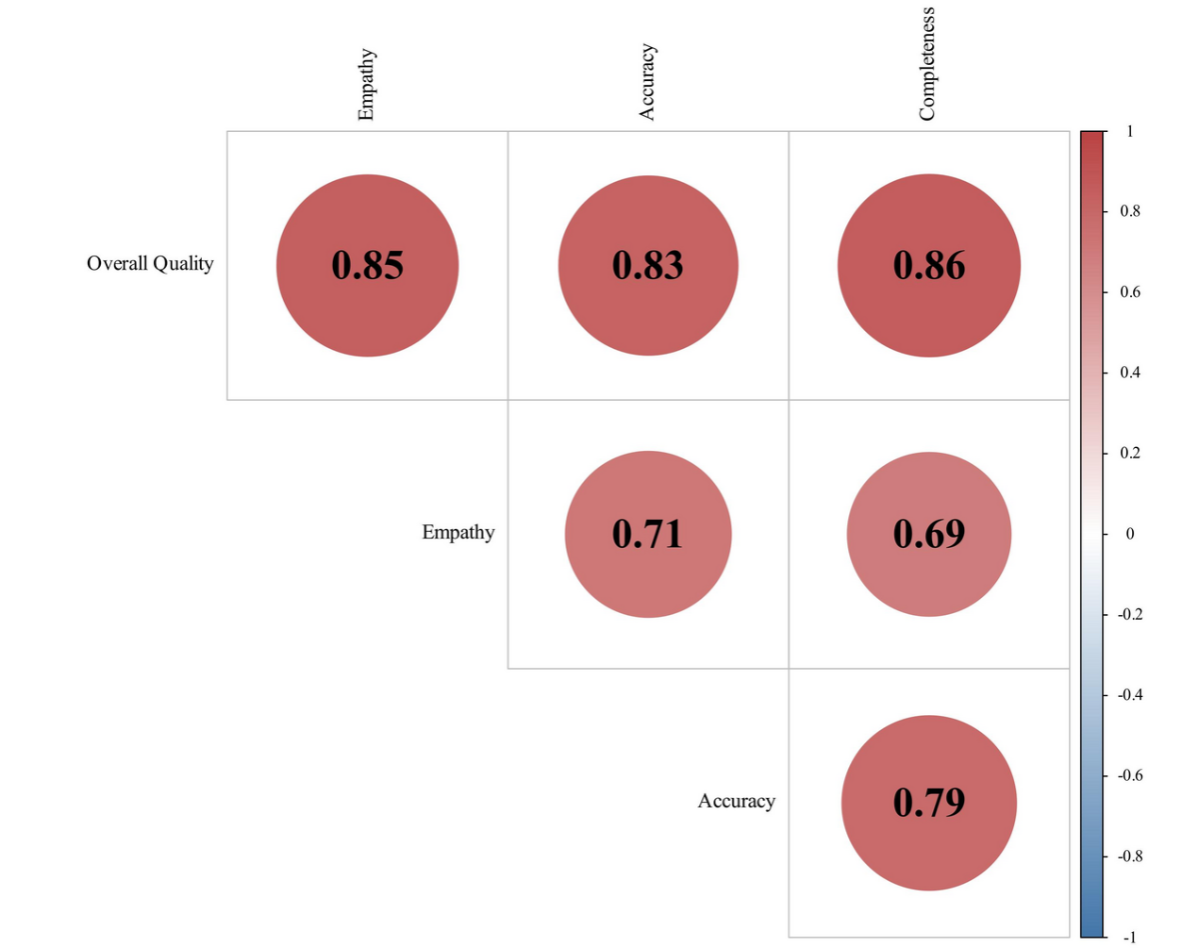
Correlation analysis of doctors' responses.

Using Pearson tests, a correlation analysis was conducted on the scores of ChatGPT responses across different text dimensions, revealing strong correlations between overall quality and other subdimensions (with empathy: r=0.842; with accuracy: r=0.839; with completeness: r=0.795). Additionally, there was a high correlation between accuracy and completeness among subdimensions (r=0.762; Figure 5). Similar patterns were observed in text responses from doctors, where overall quality exhibited correlations with completeness (r=0.857), with empathy (r=0.849), and with accuracy (0.828), and a correlation existed between accuracy and completeness (r=0.785; Figure 6).

### Readability Analysis

**Figure 7:**
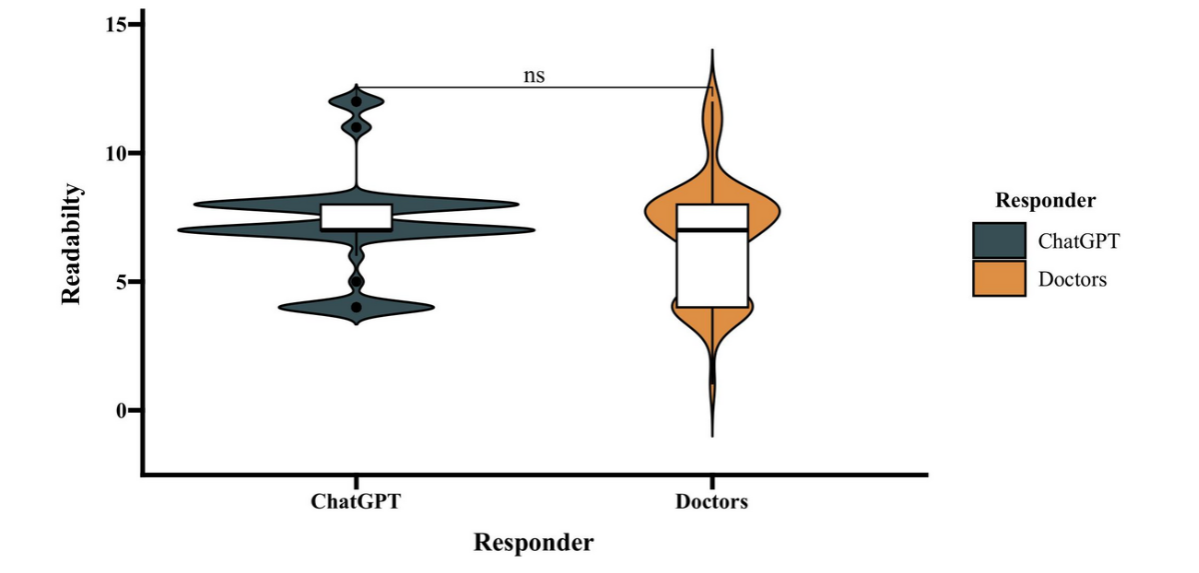
An accordion plot illustrating significant fluctuations in the text editing abilities of the original doctors’ responses (median: 7th grade; 1st quarter: 4th grade; 3rd quarter: 8th grade).

A Mann-Whitney *U* test on the readability of responses from doctors (median: 7th grade; 1st quartile: 4th grade; 3rd quartile: 8th grade) and ChatGPT (median: 7th grade; 1st quartile: 7th grade; 3rd quartile: 8th grade) revealed no significant difference (*P*=.09; Figure 7).

## Discussion

### Principal Findings

We introduced relatively novel methodologies and tools to form a viable framework for health information assessment. This cross-sectional study hypothesized that, in vertical fields like IBD, foundational LLMs such as ChatGPT-3.5 can perform comparably to domain experts in drafting health information and educating patients. Preliminary results suggest that using AI to assist with drafting or refining educational materials in patient education and popular science scenarios outside clinical consultations holds significant potential. This approach can address the challenges of limited physician time or expertise and bridge the gap of static information failing to meet the needs of patients with IBD. However, due to the current inability to eliminate AI hallucination phenomena, no matter how well LLMs perform, it is crucial to avoid unsupervised direct integration into patient care processes.

### Stable and Comprehensive: ChatGPT’s Health Information Output Capability

High-quality health information contributes to favorable medical outcomes, especially for patients with chronic conditions [12]. Conversely, erroneous, incomplete, and unregulated information may mislead patients into making detrimental choices [13]. Exploring the application of LLMs in health information retrieval and chronic disease patient education holds significant practical relevance.

Although the selection ratio did not exceed that of doctors to a statistically significant level, it is important to note that the latter are highly specialized professionals in the field of Chinese IBD and are endorsed by the expert association (Chinese IBD board) that follows them.

However, ChatGPT’s responses are more comprehensive. From a selection of 12 fully optimized ChatGPT responses (partially referenced in Multimedia Appendix 3), the advantages of ChatGPT are evident. Moreover, in the 5-level Likert scale evaluation, it significantly outperformed human experts. This stems from GPT’s structured approach based on preset models, featuring a brief introductory paragraph, followed by a list of answers with bullet points or numbering, and a standard concluding paragraph. In terms of accuracy and empathy, there were no significant differences between the two; however, there was a significant disparity in the completeness of responses between humans and GPT. Previous studies have found that GPT provides appropriate and easily understandable answers to questions regarding diagnosis and treatment choices but falls short when it comes to explaining diagnostic tests and recommending complex management strategies [7]. Although its responses are structurally sound, they often lack critical insights into decision thresholds and treatment timing [23]. Our IBD specialist physician (YC) provided a sharp critique: Its answers are superficial and lack sensitivity and understanding of medication efficacy and monitoring time frames. Interestingly, despite our blind randomized process, 2 evaluators admitted toward the end of the experiment that they could discern distinctly different styles between the 2 groups. Our study does not seem to support ChatGPT’s tendency to offer technically correct but insufficient textual conclusions [23].

From various visual distribution charts (Figures 2, 3, 4, and 6), it is evident that ChatGPT exhibits less score variance, indicating a more stable performance in this dimension. If you have ever coordinated a large group of people, compiling group publications and information without being able to control the format, you would deeply appreciate the commendable ability of LLMs in this regard. With an equal amount of learning material provided, machines produce more consistent outputs than humans.

### How to Make Others Understand (Whether They Are LLMs or Patients)

In its official description, ChatGPT is merely a language model, pretrained for general cognitive tasks [10]. Its performance may decrease when faced with tasks that require specialized and highly professional skills. Subsequent strategies include (1) using secondary LLMs tailored for various professional scenarios, such as Med-PalM [33], and (2) application of prompt engineering.

Prompt engineering, a concept that combines artistry and science [10], led to a sudden realization in our experimental design, respecting the working principles of ChatGPT. LLMs are based on large-scale learning, reflecting the collective knowledge level of most learnable materials, implying that its understanding of information is based on widely applicable domains. However, our questions were based on the IBD community, sourcing information from a vertically specialized field. Specific contexts give rise to “slang” and a plethora of “terminology.” Broadly speaking, ChatGPT interpreting [激素] “hormones” as “chemical messengers between cells” is the most accurate, as outside of clinical contexts, such abbreviations are rarely used. When faced with unsatisfactory responses or doubts about AI hallucination, consider first whether the prompts (terminology) you provide as the initiating party have been broken down for laypersons to understand. In previous evaluations of English and Chinese IBD information [15,16,34], the readability levels of web health information were generally too high, making them unsuitable for public dissemination [14]. Simultaneously, there are also articles indicating that the English output generated by ChatGPT is at a university level [9]. This aligns with the common complaint heard by the authors in work settings from patients: Doctors chatter on, but I can’t understand a word they're saying. Explaining one term with another is not a joke but a satirical reality.

In English health information research, readability analysis is commonplace. Common assessment tools include the Flesch-Reading Ease score and Flesch-Kincaid Grade Level [15,16,22,25]. However, the analysis and application of Chinese readability are still in their infancy. We hope to see more experts from various industries, not just health care professionals, participate in such research and recognize the importance of information and its potential power. The popularity of “Q&A on Ulcerative Colitis and Crohn's Disease” in the IBD community remains inexplicable, but we speculate that its readability matches the general educational levels of the Chinese population and the recommended grade level for popular science publications [35]. Additionally, it is pleasantly surprising that the Chinese readability of ChatGPT’s response information is also very good, showing no significant difference from the level of professional doctors and exhibiting greater stability (narrower kernel density variance).

In the cross-sectional comparison of subdimensions, we observed strong correlations between the overall quality of health information and completeness, accuracy, and empathy. Furthermore, there was a high predictive function between completeness and accuracy, as depicted in Figures 5 and 6, with a “more words, more reason” phenomenon. This same trend is confirmed in sensitivity analysis [21]. Although each aspect can enhance the persuasiveness of textual information, empathy as an emotional dimension is not strongly correlated with rational dimensions such as accuracy and completeness.

### Disparities in Cognitive Understanding Between Patients and Health Care Providers

In the overall comprehension of quality and completeness, it is evident that there was no disagreement among assessors in the roles of health care providers and patients. Both parties unanimously considered ChatGPT and medical experts to perform similarly, with the former providing more comprehensive information. However, upon conducting subgroup analysis, we discovered that health care providers have a delayed grasp on empathy and are more sensitive to accuracy. Health care providers can discern more accurately sourced information from their peers, while patients may not. These disparities form the foundation for the communication conflicts between health care providers and patients in real-life scenarios. Patients may not perceive ChatGPT’s information to be more erroneous than that of medical professionals, possibly due to their lack of professional knowledge to comprehend the underlying facts. This mirrors the headache-inducing situation for health care providers when patients prefer to believe exaggerated television advertisements for health products rather than opting for industry-reviewed experts and guidelines. This serves as a reminder that medical and health information must be developed and tested with patients (consumers) at the center [36].

### AI Hallucination

Errors in responses from LLMs are referred to as “AI hallucination,” and chatbots typically present themselves in a convincing manner, leading the inquirer to potentially believe in their authenticity [6,10]. We believe this is also a key reason why patient assessors cannot differentiate between the accuracy of ChatGPT and medical experts.

**Figure 8:**
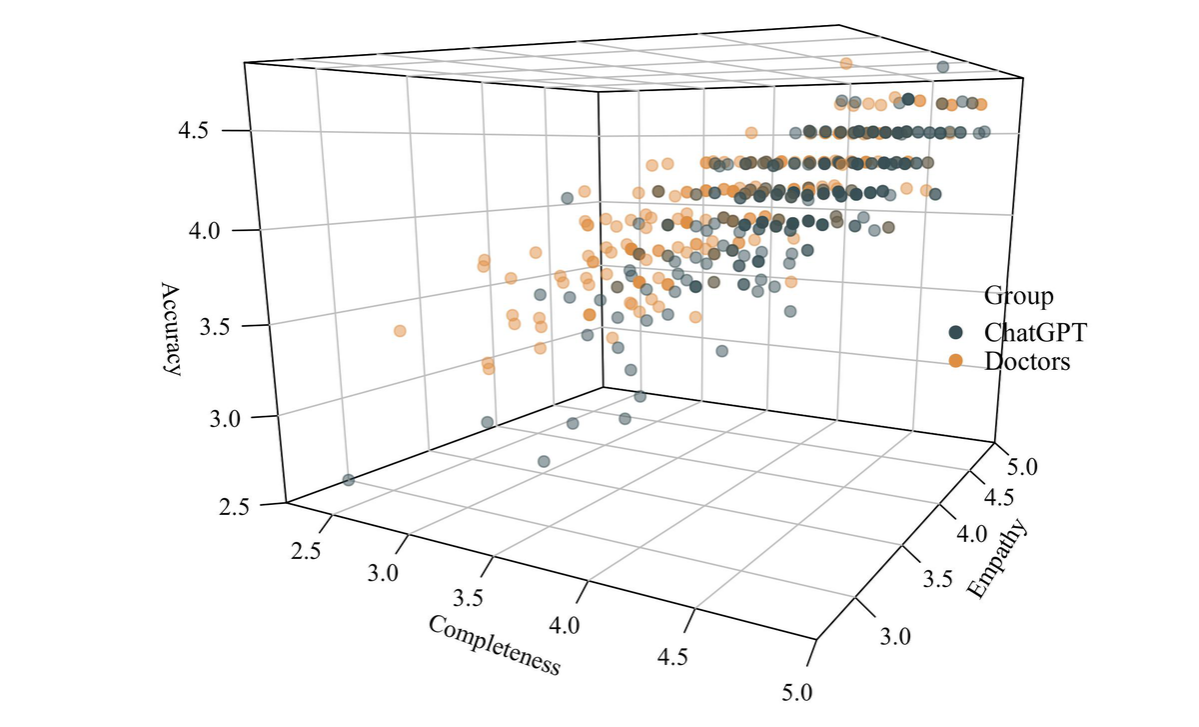
The 3D scatter plot of the dimensions, showing a few instances of extremely low responses by ChatGPT on the left side of the plot and dense distribution of ChatGPT responses on the high completeness dimension.

Despite emphasizing the importance of prompt engineering, we are still amazed by ChatGPT’s ability to identify spelling errors, ambiguities, and highly condensed issues, based on our experimental responses structured as progressive inquiries following textbook content. As feedback, ChatGPT even comprehends outdated drug translations (eg, the new official translation [英夫利西单抗] for “infliximab” and the old term [英孚利昔单抗]). It also gave us a few “AI hallucinations” (correspondingly, numerous poorly performing outliers are evident in Figure 8), where commonly used drug names in clinical practice were interpreted as the scientific names of mosquitoes and, when questioned further, ChatGPT refused to acknowledge the error (Multimedia Appendix 4). We attribute the causes of these AI delusions to a lack of background knowledge and insufficient prompts.

In the realm of medicine, a discipline that relentlessly pursues zero errors as a necessity of natural science and ethics, allowing AI to engage in self-expression is inappropriate. Therefore, we also agree that it is imperative for professionals to verify the output of ChatGPT [10,37,38], despite our observations indicating that it often performs at a level comparable to that of experts.

### Evaluating the Achievements of AI Should First Be Based on How Humans Assess Their Own Accomplishments

Although ChatGPT may provide outdated or incorrect information, the level at which an LLM operates is a key consideration. Care should be taken when comparing ChatGPT with various experts or professional guidelines. Additionally, we must consider whether our human experts are capable of effectively dissecting and conveying complex, obscure, and uncommon terms and concepts to laypersons [39]. Criticisms and warnings about LLMs are prevalent, reminding us of the need to contemplate the baseline definition of medical practice. Questions arise as to whether outputs need to strictly adhere to guidelines and if the discrepancies in guidelines among different countries, regions, and medical associations have been fully addressed. If not, evaluating LLMs or AI outputs will always involve subjective differences.

Based on our findings, we cautiously endorse the view that ChatGPT has the potential to improve patients’ access to disease information in health care settings [7,23]. Its performance may be even better when assessed by nonspecialist doctors or young medical students. If we were to compare humans to AI in the context of online community doctors, we speculate that the positive outcomes would be significantly pronounced [21].

### Next Steps in Exploration

To our knowledge, this study represents the first invitation for IBD health information consumers and providers to participate in a crowdsourced evaluation. It is also an exploration of the readability of simplified Chinese characters in the context of IBD.

Introducing tools like ChatGPT into patient communities and basic patient education settings in a timely manner seems feasible: Initiating the use of ChatGPT to draft medical information for health care providers (health self-media practitioners, health care professionals, medical institution promoters), followed by expert review and refinement, appears to be a viable and convenient production pathway. Undoubtedly, the quality of ChatGPT’s responses will gradually improve with version updates and over time, making the tool even more promising [40].

Although not the primary hypothesis of our experiment, we also observed variations in text quality between different disease types generated by ChatGPT [37]. Furthermore, ChatGPT has an overwhelming advantage over human experts in terms of speed of content creation. Many participating doctors acknowledge that crafting understandable content for patients in health education efforts requires significant dedication and effort [41].

Previously, on social media platforms and in online medical consultation scenarios, ChatGPT’s response capabilities have surpassed those of ordinary doctors in addressing common disease symptoms [21]. However, in this study’s specialized vertical field (specifically referring to IBD specialization), professional doctors still demonstrate superior judgment and threshold control in information decision-making. We can speculate that LLMs have critical threshold points in disseminating information in specialized vertical fields. It is essential for us to identify these thresholds rationally: disseminating information to laypersons below the threshold and utilizing tools to assist professionals above the threshold.

We envision a brighter future in health care, advocating for outstanding organizations (such as national cancer research centers or high-quality industry databases) to promote the dissemination of untainted high-quality data through independent reviews and exploration and subsequently leveraging digital tools like LLMs to share these data freely or affordably with patients, their families, and young doctors in need of accessing such information [4,39].

### Limitations

#### Tool and Method Selection

In order to achieve a sufficient sample size for significant effects, we temporarily set aside the assessment of evaluator consistency and well-validated information tools such as Patient Education Materials Assessment Tool and DISCERN (not disregarding them) [42,43]. Compared with subjective crowdsourced rating strategies, these questionnaires or systems have relatively higher thresholds and specific use cases. Some researchers have suggested that certain health information assessment tools may not be universally suitable [22]. If resources permit and the context is appropriate, we also recommend considering the simultaneous use of the aforementioned tools in the future and, when necessary, conducting accuracy assessments based on medical guidelines for evidence-based evaluation [23].

#### Reply Randomness and Answer Reproducibility

Many researchers argue that a key limitation of the application and reproducibility of large-scale language models lies in the inherent randomness of their generated responses [6]. This inherent randomness refers to the unpredictability of these models, primarily because they are trained on various text data and use probabilistic algorithms to generate answers. Even with multiple inputs of the same or similar content, this inherent randomness can lead to variations in the quality and accuracy of the outputs [5,7]. However, some experiments suggest that repeating questions to ChatGPT multiple times results in excellent consistency of answers, reaching 90.48% to 100% [9,23]. Our preliminary findings also indicate that, if the prompts are the same, although not identical in every aspect, the structure and substantive content of the responses from ChatGPT are generally similar (Multimedia Appendix 5). Despite the aforementioned good reproducibility of ChatGPT, we have not yet fully overcome this limitation in our experiment.

#### Model Version Changes

The use of LLMs in patient education represents an interdisciplinary field at the intersection of medicine and technology. Large-scale AI language models possess the capability for improvement and learning, rendering similar research findings potentially outdated in a short span of time [9,10,40]. Just as we completed the compilation of responses in the second week, OpenAI released a new version. This is why we adhere to an open science approach, using publicly available and traceable materials as the textual sources for this comparison. Given improved conditions, we suggest that peers could build upon this foundation to conduct more comprehensive experiments or expand them into randomized controlled trials.

#### Network Latency and Restrictions

The blocking of ChatGPT in specific regions’ IP addresses (such as mainland China and Hong Kong) has added additional challenges to the use of this technology. High latency and instances of crashes have made the entire response process lengthy. Although these limitations indeed exist, they were not reflected in our results. Exceptional technology not only guides outstanding experimental outcomes but also relies on the accessibility and low barriers to entry of that technology. It is hoped that, in the future, all individuals, especially those in underdeveloped regions, can benefit from this technology. Gratitude is extended to OpenAI and ChatGPT 3.5 for their free and open-source demonstration of the allure of LLMs and the exploration of their application scenarios.

As of the date of the revised manuscript, this limitation has been alleviated, with numerous outstanding general and specialized LLMs emerging in both China and the United States (eg, Baichuan, Qwen, LLaMA, Claude). Meanwhile, the use of ChatGPT-like alternative products for medical consultations and learning in broader health-related scenarios has gradually begun to be integrated into daily life.

#### Current Limitations in Use Scenarios

Countries worldwide have enacted citizen health information privacy protection measures [35], such as China’s Personal Information Protection Law and the United States Health Insurance Portability and Accountability Act. Under current circumstances, we cannot and do not recommend researchers directly extract patient questions from patient communities, communication social media platforms (such as WeChat, WhatsApp), or outpatient settings to pose queries to ChatGPT. This is why, when considering the adoption of patient question sources, we collect authorized publications with management oversight.

### Conclusions

In all dimensions, regardless of subjective or objective evaluation, ChatGPT demonstrated greater stability than human experts. When it came to responses to specialized medical questions, ChatGPT’s overall performance was on par with that of human specialist doctors. Its output of health information exhibited a better structural coherence, addressing the differentiation in outputs caused by cognitive and knowledge variations among individual specialist doctors. Using ChatGPT-3.5 to draft patient education materials, with doctors refining, supplementing, and proofreading the information, is acceptable and worth promoting. However, direct patient consultations and health education using ChatGPT are not feasible due to the presence of AI hallucinations. Differences in empathy and accuracy may exist between health care providers and patients. As primary consumers of health information, patients need to be involved in the creation and evaluation of health information. Before extensively applying LLMs in medical practice, more clinical trials and case studies are needed to assess their effectiveness and potential side effects. Ethical and privacy concerns, user training and education, and ongoing monitoring and evaluation are all issues that we need to consider and carefully deliberate.

## Appendix

Multimedia Appendix 1 Bilingual table of contents for Q&A on Ulcerative Colitis and Crohn's Diseas

Multimedia Appendix 2 Co-authored original responses by doctors and patients.

Multimedia Appendix 3 Selected evaluation cases of responses.

Multimedia Appendix 4 Examples of AI hallucinations.

Multimedia Appendix 5 Examples of reproducibility responses.

## Abbreviations

AI: artificial intelligence
CCCF: China Crohn’s and Colitis Foundation
CRIE: Chinese Readability Index Explorer
IBD: inflammatory bowel disease
LLM: large language model
