# Supplementary material for: Ability of ChatGPT to Replace Doctors in Patient Education: Cross-Sectional Comparative Analysis of Inflammatory Bowel Disease
Source: J Med Internet Res. 2025 Mar 31;27:e62857. doi: 10.2196/62857 (PMC11997527; doi:10.2196/62857)
Supplement: Multimedia Appendix 1 [file jmir_v27i1e62857_app1.docx]

第一章　炎症性肠病概况

一、病　因

问题1：我为什么会得克罗恩病这个奇怪的病？

问题2：炎症性肠病是否属于过敏？

二、临床表现

问题3：溃疡性结肠炎和克罗恩病的临床表现有哪些呢？

问题4：我皮肤上出现一些结节，是否可能与溃疡性结肠炎或克罗恩病有关？

三、诊　断

问题5：为什么做了那么多检查，花了那么长时间还是不能确诊呢？

问题6：听说胶囊内镜很好，它对克罗恩病的诊断意义应该很大吧？

问题7：溃疡性结肠炎和普通肠炎的区别是什么呢？

问题8：之前考虑我得的是溃疡性结肠炎，为什么后来又认为我得的是克罗恩病呢？

问题9：医生怀疑我得的是克罗恩病，需要做哪些常规检查呢？

问题10：医生说我的疾病需要与结核区别，那么如何区别结核和克罗恩病呢？

问题11：医生说我的疾病无法和结核区别，需要诊断性抗结核治疗，为什么要进行诊断性抗结核治疗？

问题12：克罗恩病的分型是怎样的？是不是有梗阻型、溃疡型这样的区分？

问题13：医生说我是“回末型”克罗恩病，请问这是什么意思呢？

四、并发症

问题14：溃疡性结肠炎的并发症有哪些？具体有什么表现？

问题15：克罗恩病的并发症有哪些？具体有什么表现？什么时候需要怀疑我出现了并发症？

五、检　查

问题16：CTE和MRE如何做？区别在哪里？

问题17：内镜检查包括哪些？需要我准备什么？

问题18：B超有什么价值？为什么有时候要穿刺？

问题19：炎症性肠病患者为什么要行肠道超声检查？该检查有什么优点？

问题20：肠道超声检查与肠镜、CT或磁共振相比有什么优缺点？

问题21：为什么我做了多次内镜检查，也做了很多病理检查，仍然无法确诊？病理检查有用吗？

六、治　疗

（一）美沙啦嗪篇

问题22：美沙拉嗪是什么？

问题23：美沙拉嗪适合治疗什么疾病？

问题24：美沙拉嗪需要服用多久？

问题25：美沙拉嗪有哪些副作用？

问题26：美沙拉嗪什么时候吃？（餐前，餐后，还是餐时？）

问题27：艾迪莎和颇得斯安在哪里释放？原理是什么？

（二）激素和免疫抑制剂篇

问题28：激素是什么？

问题29：激素有哪些作用？

问题30：激素可以治疗哪些疾病？

问题31：激素在炎症性肠病治疗中的使用剂量和疗程如何？

问题32：激素有哪些副作用？

问题33：激素用量减不下来怎么办？

问题34：免疫抑制剂是什么？

问题35：在炎症性肠病中，免疫抑制剂使用的适应证有哪些？

问题36：嘌呤类免疫抑制剂如何使用？

问题37：嘌呤类免疫抑制剂的主要副作用有哪些？

问题38：如何监测硫唑嘌呤的副作用？

问题39：使用嘌呤类免疫抑制剂导致白细胞水平降低，我该怎么办？

问题40：炎症性肠病患者为什么可以用沙利度胺，有效剂量是多少？

问题41：沙利度胺有哪些副作用？沙利度胺用于治疗炎症性肠病女性患者时需要注意哪些事情？

（三）生物制剂篇

问题42：英孚利昔是什么？

问题43：英孚利昔单抗的治疗效果如何？

问题44：哪些克罗恩病患者需要使用英孚利昔单抗进行治疗？

问题45：哪些克罗恩病患者可能需要患病早期使用英孚利昔单抗治疗（早期积极治疗） ？

问题46：采用英孚利昔单抗治疗有副作用吗？

问题47：英孚利昔单抗可以长期使用吗？

问题48：停用英孚利昔单抗后，若疾病复发，治疗还有效吗？

（四）肠内（肠外）营养篇

问题49：肠内营养可以治疗克罗恩病吗？

问题50：肠内营养摄入剂量不同对改善疾病情况的作用一样吗？

问题51：口服和鼻饲，哪种方式进行肠内营养效果更好呢？

问题52：什么情况下可能更需要口服营养补充？

（五）新的治疗方法篇

问题53：白细胞洗涤有用吗？

问题54：粪菌移植有用吗？

问题55：自身免疫疗法有用吗？

问题56：副结核分枝杆菌（MAP）与克罗恩病有什么关系？

（六）特定碳水化合物治疗篇

问题57：用特定碳水化合物治疗炎症性肠病是否可行？

（七）内镜治疗篇

问题58：什么是内镜治疗？

问题59：克罗恩病患者在哪些情况下可以进行内镜治疗？（克罗恩病内镜治疗的适应证有哪些？）

问题60：克罗恩病并发消化道出血时，如何进行内镜治疗？

问题61：克罗恩病并发肠梗阻时，如何进行内镜治疗？

问题62：克罗恩病并发瘘管时，如何进行内镜治疗？

问题63：克罗恩病并发腹腔脓肿时，如何进行内镜治疗？

（八）随访和复发

问题64：随访是什么？

问题65：对炎症性肠病患者，随访内容一般有哪些？

问题66：炎症性肠病是一种需要长期治疗的慢性疾病，可能会伴随您的一生，那么如何预防它的复发呢？

问题67：患者应注意哪些方面，如何判断病情是加重还是减轻？

问题68：患者自己如何判断肚子痛是由梗阻引起的，还是由吃了不洁食物所致的？

问题69：炎症性肠病患者腹泻时应该怎么办？

问题70：炎症性肠病的辅助药物有哪些？

问题71：指标好的患者如何发现炎症性肠病是否活动或复发？

问题72：炎症性肠病患者在什么情况下可以停药？

（九）手术治疗

问题73：为什么我需要手术？

问题74：什么时候合适手术？

问题75：手术准备需要注意什么？

问题76：医生会选用什么手术方式？

问题77：怎么判断是“开大刀”手术，还是腹腔镜手术？

问题78：患了克罗恩病，又患“痔疮”，怎么办？

问题79：溃疡性结肠炎需要手术，我又有部分结肠是正常的，能不能只切除有病变的肠管呢？

问题80：患了克罗恩病，又患“肛裂”，怎么办？

问题81：什么是瘘？瘘都有哪些类型？一般有什么表现？怎么检查是否有瘘？怎么处理？

问题82：炎症性肠病女性患者手术会对生育有影响吗？男性呢？

问题83：什么是择期手术和急诊手术？

问题84：肠梗阻时，是不是一定要马上手术？有没有其他的保守治疗方法？

问题85：什么是包块？包块危险吗？包块一般如何处理？有包块是不是一定要手术？有没有办法可以不手术？

问题86：术后康复要注意什么？术后多久可以下地行走？

问题87：造口护理有什么注意事项？

问题88：病变累及肛门导致肛门狭窄，如何扩肛？

问题89：病变累及肛门导致肛门狭窄，如何灌肠？

问题90：哪些药物可以用于炎症性肠病灌肠？

问题91：炎症性肠病患者如何使用肛门栓剂呢？

问题92：用于炎症性肠病的肛门栓剂有哪些呢？

问题93：炎症性肠病患者如何坐浴呢？

问题94：炎症性肠病患者用于坐浴的药物有哪些呢？

问题95：如果得了肛周疾病，如何进行护理呢？

第二章　中西医结合治疗

问题96：能否采用中医中药治疗炎症性肠病？

问题97：炎症性肠病患者进行理疗，可行吗？锻炼、按摩、针灸、艾灸、泡脚等又有何利与弊？

问题98：中医能治愈炎症性肠病吗？

第三章　癌变问题

问题99：“如果有增生是否就完蛋了？”——高危和癌变问题

问题100：如何检测癌变？

第四章　饮　食

一、饮食与炎症性肠病发病、症状、治疗

问题101：我的炎症性肠病是饮食引起的吗？

问题102：饮食与我的炎症性肠病症状有关吗？

问题103：食物能够治愈我的病吗？

问题104：特定碳水化合物饮食能够治愈我的病吗？

问题105：草药能够治愈我的病吗？

二、消化与营养

问题106：食物在人体内是如何消化的？

问题107：我得了炎症性肠病，它是如何影响我消化食物的？

问题108：营养物质在人体内有什么作用？

问题109：我的肠道还能正常吸收营养物质吗？

问题110：为什么得了炎症性肠病（尤其是克罗恩病）让我更易出现营养问题？

三、个体化饮食建议

问题111：是否有适合我们患者的某种饮食？

问题112：作为炎症性肠病患者，我应该避免哪些食物？

问题113：我应该如何进行个体化饮食？

问题114：我应该如何记录饮食日记？

四、炎症性肠病管理可能涉及的概念

问题115：作为炎症性肠病患者，我可能涉及的饮食类型有哪些？

问题116：为促进疾病管理，我有哪些需要了解的饮食概念呢？可以解释一下低渣饮食等概念吗？

问题117：是否存在我们患者需要区别的一些饮食概念？

五、病情不同阶段的饮食建议

问题118：我目前处于疾病缓解期，该如何饮食呢？

问题119：我目前处于疾病发作期，该如何饮食呢？

问题120：腹泻时，我该如何饮食呢？

问题121：腹痛、腹泻时，我可以尝试的食物有哪些？

问题122：腹痛、腹泻时，我可能需要避免的食物有哪些？

问题123：炎症性肠病逐渐好转，我需要改变饮食吗？

六、炎症性肠病患者相应症状或病变的饮食建议

问题124：我便秘了，怎么办？

问题125：我该如何保持正常体重？

问题126：医师说我有肠道狭窄，我的饮食应该注意什么？

七、炎症性肠病患者特殊情况下的营养补充及饮食建议

问题127：我打算怀孕，关于怀孕的营养补充有哪些建议呢？

问题128：作为儿童或青少年患者，我的营养补充要注意什么？

问题129：作为回肠切除患者，我的营养补充要注意什么？

问题130：作为造口患者，我的营养补充要注意什么？

问题131：作为短肠综合征患者，我的营养补充要注意什么？

八、可能让您左右为难的问题——食物方面

问题132：我能喝牛奶吗？

问题133：我是乳糖不耐受患者，我还能喝牛奶吗？

问题134：我可以喝酸奶吗？

问题135：我能吃水果吗？

问题136：我能喝鸡汤吗？

问题137：我可以食用海鲜食品吗？

问题138：我能喝饮料吗？

问题139：我能吃零食吗？

问题140：我能喝茶吗？

九、额外营养补充

问题141：我需要补充维生素吗？

问题142：我需要补铁吗？

问题143：我该如何补铁呢？

问题144：我需要补钙吗？

问题145：我该如何补钙呢？

问题146：我需要补充维生素D吗？

问题147：我该如何补充维生素D呢？

问题148：我需要补充叶酸吗？

问题149：我需要补充维生素B12吗？

问题150：我需要补充鱼油吗？

问题151：我该如何补充ω-3多不饱和脂肪酸呢？

问题152：我服用益生菌有好处吗？

问题153：我服用益生元有好处吗？

问题154：我服用合生元有好处吗？

问题155：我可以吃人参、灵芝、膏方、蜂王浆、铁皮枫斗等营养品吗？

十、可能让您不知所措的问题

问题156：我老是要放屁，怎么办？

问题157：我该如何找到可能使自己放屁增多的食物？

问题158：外出聚餐时，我该如何饮食呢？

问题159：节假日时，我该如何饮食呢？

问题160：我喜欢吃比萨饼、冰淇淋、汉堡包等这类食物。得了炎症性肠病，我是不是完全不能再吃了？

问题161：一位有经验的病友告诉我他不能吃某类食物，我是不是也应该避免吃这类食物呢？

问题162：为了安全起见，我是不是应该尽量不吃一些可能有问题的食物，如牛奶？

参考文献

第五章　运动和生活

问题163：我是克罗恩病患者，目前病情稳定，哪些运动适合像我这样的患者？

问题164：免疫接种（疫苗）是什么东西？

问题165：炎症性肠病患者为什么要关注免疫接种（疫苗） ？与正常人有什么不同？

问题166：炎症性肠病患者应该接受哪些免疫接种？

问题167：那该在什么时候进行免疫接种呢？

问题168：哪些疫苗是安全的？

问题169：我在使用免疫抑制剂过程中，突然被狗或猫咬了怎么办？

问题170：我在使用免疫抑制剂过程中，需要打破伤风疫苗怎么办？

问题171：妊娠期女性注射疫苗有什么注意事项？

问题172：炎症性肠病女性患者妊娠期间药物的使用对新生儿免疫接种的影响如何？

问题173：关于养宠物与疫苗问题：IBD患者可以打疫苗吗？可否养宠物？被宠物咬了怎么办？

问题174：有炎症性肠病的，再患其他疾病怎么处理？比如感冒、发烧了怎么办？用治疗这些病的药对炎症性肠病的病情有影响吗？

问题175：眼睛红了怎么办？是并发症呢，还是其他原因？

问题176：炎症性肠病患者可以用抗生素吗？怎么对待抗生素？

问题177：炎症性肠病患者可否运动、上班、工作、学习？

问题178：炎症性肠病患者出门探亲、旅游的注意事项和如何应对突发情况？

参考文献

第六章　特殊情况下的注意事项

问题179：炎症性肠病患者应该因为患病而选择放弃生育吗？

问题180：女性炎症性肠病患者怀孕时能不能用药？

问题181： 怀孕时疾病处于活动期或手术治疗会导致不良的妊娠后果么？

问题182：我是一名克罗恩病女性患者，打算要孩子，需要注意些什么？是不是C反应蛋白（CRP）、血沉等炎症指标好就可以了？

问题183：我现在怀孕了，怕药物影响胎儿，就停用了所有药物，请问这样可以吗？

问题184：我顺利生了孩子，现在在服用治疗克罗恩病的药物，我可以哺乳吗？

问题185：男性患者的生育问题——什么时候可以生育，停不停药？

问题186：担心自己的孩子也得这个疾病，对自己的孩子，如何更好地做好预防工作？

第七章　儿童和青少年问题

一、致儿童及青少年

问题187：我得了克罗恩病，感觉压力很大，常常睡不好，我该怎么办？

问题188：这种疾病会不会影响我的身高？

问题189：什么治疗方法比较适合我？

问题190：因为经常肚子痛想上厕所，我有点害怕去公共场所（如剧院、超市、商场、车站等）、去旅游或去学校，怎么办？

问题191：我可以参加体育运动吗？

二、致家长

问题192：作为家长，我做什么能让生活变得更好？

问题193：我怎么知道我的孩子生长发育有问题？

问题194：青春期延迟是怎样的？我的小孩是正常的吗？

问题195：怎样教育孩子独立处理疾病带来的麻烦？

问题196：我怎样才能让我的孩子保持一个积极的态度来对待疾病？

问题197：我要向学校老师说明孩子的情况吗？

问题198：我能做什么来帮助我的孩子读好书？

第八章　生活习惯

一、吸烟与炎症性肠病发病

问题199：我得了溃疡性结肠炎，是不是吸烟引起的？

问题200：我得了克罗恩病，是不是吸烟引起的？

二、吸烟与炎症性肠病病情

问题201：我是溃疡性结肠炎患者，吸烟是怎样影响我的病情的？

问题202：我是克罗恩病患者，吸烟是怎样影响我的病情的？

问题203：我是克罗恩病患者，每天只吸3支烟应该没影响吧？

问题204：为什么吸烟对克罗恩病和溃疡性结肠炎的影响这么不同呢？

三、被动吸烟与炎症性肠病

问题205：被动吸烟（吸二手烟）与我得溃疡性结肠炎及疾病的症状有关吗？

问题206：被动吸烟（吸二手烟）与我得克罗恩病及疾病的症状有关吗？

四、戒　烟

问题207：我是溃疡性结肠炎患者，戒烟值得吗？

问题208：作为吸烟的溃疡性结肠炎患者，我戒烟后疾病会加重吗？

问题209：听说尼古丁能够治疗溃疡性结肠炎，我能使用这种方法吗？

问题210：我是吸烟的克罗恩病患者，戒烟值得吗？

问题211：我努力尝试戒烟，但发现很难，有没有什么方法能够帮助我戒烟？

五、饮　酒

问题212：饮酒会导致我疾病复发吗？

问题213：我饮酒后会有不适吗？

问题214：我需要限制饮酒吗？

问题215：我在服药期间能够饮酒吗？

问题216：当我疾病稳定时，选择哪种酒更合适呢？

问题217：每天适度饮酒对我来说合适吗？

六、压力管理

问题218：我感到压力大时，疾病似乎更严重。压力与炎症性肠病有关吗？

问题219：面对生活中的压力，我该怎样调节呢？

七、其　他

问题220：得了炎症性肠病，我还能运动锻炼吗？

问题221：我容易忘记吃药。有没有什么方法能帮助我记住按时吃药呢？

问题222：夏天外出时，我有什么需要注意的吗？

参考文献

第九章　心理问题

问题223：得病后，我心里非常苦闷，原来快乐的我不见了，我该怎么办？

问题224：我自己总是调整不好心理状态，有什么好办法吗？

问题225：当亲人被确诊为炎症性肠病后，我该怎样帮助他？

问题226：作为一名炎症性肠病患者，我要怎么去面对生活？

问题227：建立患友俱乐部的意义有哪些呢？

第十章　自我管理

一、入门篇

问题228：在网上看到许多关于克罗恩病的说法，有的说得非常可怕，是真的吗？

问题229：除了去医院问医生，我还可以从哪里了解到可靠的IBD知识？

问题230：看到药品说明书上的副作用，我感觉很害怕，怎么办？

问题231：看到那些虽然不健康但非常好吃的东西，总是控制不住去吃，怎么办？

问题232：我很瘦，但医生总让我验许多的血液项目，这会对身体不好吗？

问题233：每次的化验单上都有很多项目，哪些是我要特别关注的？

问题234：做肠镜会不会很痛？我要注意些什么？ 224

二、进阶篇

问题235：在治疗一段时间后病情有所缓解，下一步我要怎么办？

问题236：炎症性肠病患者的化验单上时常会有很多箭头，它一般反映什么？

问题237：使用硫唑嘌呤治疗后，我要怎么样监控我的血象？

问题238：有什么办法可以帮助减少药物的副作用，让白细胞、红细胞指标好一点？

问题239：听说炎症性肠病的症状和用药的个体差异都比较大，我要怎样才能获得更好的疗效？

问题240：有的伙伴在鼻饲治疗，插鼻胃管有危险吗？我可以自己尝试吗？

问题241：我并发了肛周脓肿或肛瘘，我该怎么办？

问题242：加入炎症性肠病病友的大家庭，彼此相互鼓励、相互帮助对我们来说很重要吗？

第十一章　生活质量

一、生活质量一般信息

问题243：我在看病的时候，医生和我说炎症性肠病管理的最终目标是提高生活质量。到底什么是生活质量呢？

问题244：为什么我需要特别关注生活质量？

问题245：生活质量那么重要，医护人员是怎样了解我的生活质量呢?

二、生活质量现状与影响因素

问题246：作为炎症性肠病患者，我们的生活质量是怎样的？

问题247：哪些因素可能影响我的生活质量呢？

问题248：疾病活动对生活质量影响很大，我可以通过哪些方式控制病情、促进疾病缓解呢？

问题249：是不是我得病时间越长，生活质量越差？

三、炎症性肠病对日常生活的影响

问题250：炎症性肠病对我的日常生活可能产生哪些影响呢？

问题251：我还能过正常的生活吗？

问题252：我还能正常工作吗？

问题253：我还能正常学习吗？

问题254：我还能享受性生活吗？

问题255：得了炎症性肠病，我可以外出旅游吗？

四、可能让您不知所措的问题

问题256：我还这么年轻，得了炎症性肠病会不会影响我的寿命呢？

问题257：不良的心理情绪会不会影响我的病情呢？

问题258：得了炎症性肠病，我要告诉别人吗？

问题259：有时我感到身边的人无法很好地理解我，我该怎么办？

问题260：我有时感觉得不到身边人的理解，很想与病友交流，有哪些途径可以让我接触到他们呢？

问题261：网络中有一些炎症性肠病的信息，我能尝试使用吗？

问题262：对于炎症性肠病，我有很多疑问，但就诊时常常忘记问，我该怎么办？

参考文献

第十二章　随　访

问题263：平时要检查哪些指标，多久检查一次？

Chapter 1 Overview of Inflammatory Bowel Disease

I. Causes

Question 1: Why do I have this strange disease called Crohn's disease?

Question 2: Is inflammatory bowel disease considered an allergy?

II. Clinical Manifestations

Question 3: What are the clinical manifestations of ulcerative colitis and Crohn's disease?

Question 4: I have some nodules on my skin; could they be related to ulcerative colitis or Crohn's disease?

III. Diagnosis

Question 5: Why, after so many tests and such a long time, can't a diagnosis be made?

Question 6: I heard capsule endoscopy is very effective; should it be significant in diagnosing Crohn's disease?

Question 7: What is the difference between ulcerative colitis and common enteritis?

Question 8: Initially, it was considered that I had ulcerative colitis; why was it later believed to be Crohn's disease?

Question 9: If the doctor suspects I have Crohn's disease, what routine tests should I undergo?

Question 10: The doctor mentioned my disease needs to be distinguished from tuberculosis; how can tuberculosis and Crohn's disease be differentiated?

Question 11: The doctor mentioned my disease cannot be distinguished from tuberculosis, and diagnostic anti-tuberculosis treatment is needed; why undergo diagnostic anti-tuberculosis treatment?

Question 12: What are the classifications of Crohn's disease? Are there distinctions like obstructive and ulcerative types?

Question 13: The doctor said I have "ileocecal-type" Crohn's disease; what does this mean?

IV. Complications

Question 14: What are the complications of ulcerative colitis? What are the specific manifestations?

Question 15: What are the complications of Crohn's disease? What are the specific manifestations? When should I suspect the presence of complications?

V. Examinations

Question 16: How are CTE and MRE performed? What are the differences?

Question 17: What does endoscopic examination include? What do I need to prepare?

Question 18: What is the value of ultrasound (B-mode)? Why is puncture sometimes necessary?

Question 19: Why do patients with inflammatory bowel disease need intestinal ultrasound examination? What are the advantages of this examination?

Question 20: What are the pros and cons of intestinal ultrasound compared to endoscopy, CT, or MRI?

Question 21: Why, after multiple endoscopic and pathological examinations, is a diagnosis still not confirmed? Is pathological examination useful?

Chapter 6: Treatment

(I) Mesalazine Section

Question 22: What is Mesalazine?

Question 23: What diseases is Mesalazine suitable for treating?

Question 24: How long should Mesalazine be taken?

Question 25: What are the side effects of Mesalazine?

Question 26: When should Mesalazine be taken? (Before meals, after meals, or during meals?)

Question 27: Where are Atiasa and Pentasa released, and what is the principle behind it?

(II) Corticosteroids and Immunosuppressants Section

Question 28: What are corticosteroids?

Question 29: What are the functions of corticosteroids?

Question 30: What diseases can corticosteroids treat?

Question 31: What are the dosage and duration of corticosteroid use in the treatment of inflammatory bowel disease?

Question 32: What are the side effects of corticosteroids?

Question 33: What to do if the dosage of corticosteroids cannot be reduced?

Question 34: What are immunosuppressants?

Question 35: What are the indications for the use of immunosuppressants in inflammatory bowel disease?

Question 36: How are purine immunosuppressants used?

Question 37: What are the main side effects of purine immunosuppressants?

Question 38: How to monitor the side effects of azathioprine?

Question 39: What should I do if my white blood cell level decreases due to the use of purine immunosuppressants?

Question 40: Why can inflammatory bowel disease patients use thalidomide, and what is the effective dose?

Question 41: What are the side effects of thalidomide, and what should be considered when treating female patients with inflammatory bowel disease?

(III) Biologics Section

Question 42: What is Infliximab?

Question 43: What is the therapeutic effect of Infliximab monoclonal antibody?

Question 44: Which Crohn's disease patients need to use Infliximab for treatment?

Question 45: In which early-stage Crohn's disease patients may need early aggressive treatment with Infliximab?

Question 46: Does treatment with Infliximab monoclonal antibody have side effects?

Question 47: Can Infliximab monoclonal antibody be used for a long time?

Question 48: After discontinuing Infliximab monoclonal antibody, if the disease relapses, is the treatment still effective?

(IV) Enteral (Parenteral) Nutrition Section

Question 49: Can enteral nutrition treat Crohn's disease?

Question 50: Does the effect of enteral nutrition vary with different intake doses?

Question 51: Which is more effective for enteral nutrition, oral or nasal feeding?

Question 52: Under what circumstances might oral nutritional supplementation be more needed?

(V) New Treatment Methods Section

Question 53: Is leukocyte washing effective?

Question 54: Is fecal microbiota transplantation effective?

Question 55: Is autologous immunotherapy effective?

Question 56: What is the relationship between Mycobacterium avium subspecies paratuberculosis (MAP) and Crohn's disease?

(VI) Specific Carbohydrate Diet Section

Question 57: Is the specific carbohydrate diet feasible for treating inflammatory bowel disease?

(VII) Endoscopic Treatment Section

Question 58: What is endoscopic treatment?

Question 59: Under what circumstances can Crohn's disease patients undergo endoscopic treatment? (What are the indications for endoscopic treatment in Crohn's disease?)

Question 60: How is endoscopic treatment performed when Crohn's disease complicates gastrointestinal bleeding?

Question 61: How is endoscopic treatment performed when Crohn's disease complicates intestinal obstruction?

Question 62: How is endoscopic treatment performed when Crohn's disease complicates fistula?

Question 63: How is endoscopic treatment performed when Crohn's disease complicates abdominal abscess?

(VIII) Follow-up and Relapse

Question 64: What is follow-up?

Question 65: What are the general contents of follow-up for patients with inflammatory bowel disease?

Question 66: Inflammatory bowel disease is a chronic disease that may accompany me throughout my life, so how can it be prevented from relapsing?

Question 67: What aspects should I pay attention to, and how can I judge whether the condition is worsening or improving?

Question 68: How can I determine if abdominal pain is caused by obstruction or ingestion of contaminated food?

Question 69: What should I do when I have diarrhea due to inflammatory bowel disease?

Question 70: What are the adjuvant drugs for inflammatory bowel disease?

Question 71: For patients with good indicators, how can I determine if inflammatory bowel disease is active or relapsing?

Question 72: Under what circumstances can I stop taking medication for inflammatory bowel disease?

(IX) Surgical Treatment

Question 73: Why do I need surgery?

Question 74: When is surgery appropriate?

Question 75: What should I note in surgical preparation?

Question 76: What surgical method will the doctor choose?

Question 77: How to determine whether it is "open surgery" or laparoscopic surgery?

Question 78: If I have Crohn's disease and hemorrhoids, what should I do?

Question 79: In the case of ulcerative colitis requiring surgery, and some parts of the colon are normal, can only the affected bowel be removed?

Question 80: If I have Crohn's disease and anal fissures, what should I do?

Question 81: What is a fistula? What are the types of fistulas? What are the general manifestations? How is it checked for the presence of a fistula? How is it handled?

Question 82: Does surgery for female patients with inflammatory bowel disease affect fertility? How about for males?

Question 83: What is elective surgery and emergency surgery?

Question 84: Is surgery always necessary for intestinal obstruction? Are there other conservative treatment methods?

Question 85: What is an abscess? Is an abscess dangerous? How is an abscess generally treated? Is surgery always necessary for an abscess? Is there a way to avoid surgery?

Question 86: What should I pay attention to during postoperative recovery? How long after surgery can I walk?

Question 87: What are the considerations for stoma care?

Question 88: If the lesion involves the anus, causing anal stenosis, how to dilate the anus?

Question 89: If the lesion involves the anus, causing anal stenosis, how to perform enema?

Question 90: What drugs can be used for enema in inflammatory bowel disease?

Question 91: How do patients with inflammatory bowel disease use rectal suppositories?

Question 92: What rectal suppositories are used for inflammatory bowel disease?

Question 93: How do patients with inflammatory bowel disease take sitz baths?

Question 94: What drugs are used for sitz baths in inflammatory bowel disease?

Question 95: If I have perianal disease, how should it be cared for?

Chapter 2: Integrative Treatment of Traditional Chinese and Western Medicine

Question 96: Can traditional Chinese medicine be used to treat inflammatory bowel disease?

Question 97: Is physical therapy feasible for patients with inflammatory bowel disease? What are the pros and cons of exercises, massages, acupuncture, moxibustion, foot baths, and other therapies?

Question 98: Can traditional Chinese medicine cure inflammatory bowel disease?

Chapter 3: Issues Related to Cancerization

Question 99: "If there is proliferation, is it all over?" – High-risk and cancerization issues

Question 100: How to detect cancerization?

Chapter 4: Diet

Section 1: Diet and Its Relationship with the Onset, Symptoms, and Treatment of Inflammatory Bowel Disease

Question 101: Is my inflammatory bowel disease caused by diet?

Question 102: Is my diet related to the symptoms of my inflammatory bowel disease?

Question 103: Can food cure my disease?

Question 104: Can a specific carbohydrate diet cure my disease?

Question 105: Can herbal remedies cure my disease?

Section 2: Digestion and Nutrition

Question 106: How is food digested in the human body?

Question 107: I have inflammatory bowel disease; how does it affect my digestion?

Question 108: What role do nutrients play in the human body?

Question 109: Can my intestines still absorb nutrients normally?

Question 110: Why does having inflammatory bowel disease (especially Crohn's disease) make me more prone to nutritional issues?

Section 3: Personalized Dietary Recommendations

Question 111: Is there a specific diet suitable for patients like us?

Question 112: As an inflammatory bowel disease patient, what foods should I avoid?

Question 113: How can I personalize my diet?

Question 114: How should I keep a food diary?

Section 4: Concepts Involved in Inflammatory Bowel Disease Management

Question 115: What types of diets may be relevant to me as an inflammatory bowel disease patient?

Question 116: What dietary concepts do I need to understand to promote disease management? Can you explain concepts like a low-residue diet?

Question 117: Are there dietary concepts that patients like us need to distinguish?

Section 5: Dietary Recommendations for Different Stages of the Disease

Question 118: I am currently in remission; how should I adjust my diet?

Question 119: I am currently experiencing a flare-up; how should I adjust my diet?

Question 120: What should I eat when experiencing diarrhea?

Question 121: What foods can I try when experiencing abdominal pain and diarrhea?

Question 122: What foods should I possibly avoid when experiencing abdominal pain and diarrhea?

Question 123: As my inflammatory bowel disease gradually improves, do I need to change my diet?

Section 6: Dietary Recommendations for Symptoms or Conditions in Inflammatory Bowel Disease Patients

Question 124: I am constipated; what should I do?

Question 125: How can I maintain a normal weight?

Question 126: The doctor said I have intestinal stenosis; what should I consider in my diet?

Section 7: Nutritional Supplements and Dietary Recommendations for Special Cases in Inflammatory Bowel Disease Patients

Question 127: I plan to become pregnant; what nutritional supplements do you recommend?

Question 128: As a child or adolescent patient, what should I consider in my nutritional supplements?

Question 129: As a patient with ileocecal resection, what should I consider in my nutritional supplements?

Question 130: As an ostomy patient, what should I consider in my nutritional supplements?

Question 131: As a short bowel syndrome patient, what should I consider in my nutritional supplements?

Section 8: Dilemmas Regarding Food

Question 132: Can I drink milk?

Question 133: I am lactose intolerant; can I still drink milk?

Question 134: Can I eat yogurt?

Question 135: Can I eat fruits?

Question 136: Can I have chicken soup?

Question 137: Can I consume seafood?

Question 138: Can I drink beverages?

Question 139: Can I eat snacks?

Question 140: Can I drink tea?

Section 9: Additional Nutritional Supplements

Question 141: Do I need to supplement with vitamins?

Question 142: Do I need to supplement with iron?

Question 143: How should I supplement with iron?

Question 144: Do I need to supplement with calcium?

Question 145: How should I supplement with calcium?

Question 146: Do I need to supplement with vitamin D?

Question 147: How should I supplement with vitamin D?

Question 148: Do I need to supplement with folic acid?

Question 149: Do I need to supplement with vitamin B12?

Question 150: Do I need to supplement with fish oil?

Question 151: How should I supplement with omega-3 fatty acids?

Question 152: Is there a benefit to taking probiotics?

Question 153: Is there a benefit to taking prebiotics?

Question 154: Is there a benefit to taking synbiotics?

Question 155: Can I take nutritional supplements such as ginseng, lingzhi (reishi mushroom), herbal formulas, royal jelly, or iron tree bark?

Section 10: Overwhelming Food-related Dilemmas

Question 156: I constantly have flatulence; what should I do?

Question 157: How do I identify foods that may increase flatulence?

Question 158: What should I eat when dining out?

Question 159: What should I eat during holidays?

Question 160: I love foods like pizza, ice cream, hamburgers, etc. Can I still eat them after being diagnosed with inflammatory bowel disease?

Question 161: A seasoned patient told me he cannot eat certain foods; should I also avoid those foods?

Question 162: For safety reasons, should I try to avoid certain foods, such as milk?

Question 163: As a Crohn's disease patient with stable conditions, what exercises are suitable for someone like me?

Question 164: What is immunization (vaccination)?

Question 165: Why should inflammatory bowel disease patients pay attention to immunization (vaccination)? What differences exist compared to healthy individuals?

Question 166: What immunizations should inflammatory bowel disease patients receive?

Question 167: When should immunizations be administered?

Question 168: Which vaccines are considered safe?

Question 169: What should I do if I get bitten by a dog or cat while on immunosuppressive medication?

Question 170: What should I do if I need a tetanus shot while on immunosuppressive medication?

Question 171: Are there any precautions for pregnant women receiving vaccines?

Question 172: How does the use of medication during pregnancy by female patients with inflammatory bowel disease affect the immunization of newborns?

Question 173: Regarding pets and vaccination: Can IBD patients get vaccinated? Can they have pets? What to do if bitten by a pet?

Question 174: For those with inflammatory bowel disease, how should other illnesses be managed? What if I have a cold or fever? Does using medication for these illnesses affect the condition of inflammatory bowel disease?

Question 175: What should I do if my eyes are red? Is it a complication, or could it be due to other reasons?

Question 176: Can inflammatory bowel disease patients use antibiotics? How should antibiotics be handled?

Question 177: Can inflammatory bowel disease patients engage in exercise, go to work, study, or hold a job?

Question 178: What precautions should inflammatory bowel disease patients take when traveling for family visits or tourism? How should they handle unexpected situations?

Chapter 6: Special Considerations

Question 179: Should inflammatory bowel disease patients choose to forgo having children due to the illness?

Question 180: Can female inflammatory bowel disease patients use medication during pregnancy?

Question 181: Will having the disease in an active state or undergoing surgical treatment during pregnancy lead to adverse pregnancy outcomes?

Question 182: As a female Crohn's disease patient planning to have a child, what precautions should I take? Is it enough to monitor inflammatory indicators such as C-reactive protein (CRP) and erythrocyte sedimentation rate (ESR)?

Question 183: I am currently pregnant and stopped all medications for fear of affecting the fetus. Is this okay?

Question 184: I successfully gave birth, and now I am taking medication to treat Crohn's disease. Can I breastfeed?

Question 185: Male patient fertility issues - when can they conceive, and should they stop taking medication?

Question 186: Concerned about the child inheriting the disease, how can I better prevent it in my child?

Chapter 7: Pediatric and Adolescent Issues

Section 1: Addressing Children and Adolescents

Question 187: I have Crohn's disease and often feel stressed, leading to poor sleep. What should I do?

Question 188: Will this disease affect my height?

Question 189: What treatment methods are more suitable for me?

Question 190: Because of frequent abdominal pain and the need to use the bathroom, I am a bit afraid of public places (such as theaters, supermarkets, malls, stations), traveling, or going to school. What should I do?

Question 191: Can I participate in sports?

Section 2: Addressing Parents

Question 192: As a parent, what can I do to make life better?

Question 193: How do I know if my child's growth and development are problematic?

Question 194: What is delayed puberty? Is my child normal?

Question 195: How to educate children to independently deal with the troubles caused by the disease?

Question 196: How can I help my child maintain a positive attitude towards the disease?

Question 197: Should I explain my child's condition to school teachers?

Question 198: What can I do to help my child excel in school?

Chapter 8: Lifestyle Habits

Section 1: Smoking and the Onset of Inflammatory Bowel Disease

Question 199: I have ulcerative colitis; is it caused by smoking?

Question 200: I have Crohn's disease; is it caused by smoking?

Section 2: Smoking and the Course of Inflammatory Bowel Disease

Question 201: As a patient with ulcerative colitis, how does smoking affect my condition?

Question 202: As a patient with Crohn's disease, how does smoking affect my condition?

Question 203: I'm a Crohn's disease patient and only smoke three cigarettes a day; should it have no impact?

Question 204: Why is the impact of smoking different for Crohn's disease and ulcerative colitis?

Section 3: Passive Smoking and Inflammatory Bowel Disease

Question 205: Is passive smoking (secondhand smoke) related to my ulcerative colitis and its symptoms?

Question 206: Is passive smoking (secondhand smoke) related to my Crohn's disease and its symptoms?

Section 4: Quitting Smoking

Question 207: As an ulcerative colitis patient, is it worthwhile to quit smoking?

Question 208: As a smoking ulcerative colitis patient, will my disease worsen after quitting smoking?

Question 209: I heard nicotine can treat ulcerative colitis; can I use this method?

Question 210: As a smoking Crohn's disease patient, is it worth quitting smoking?

Question 211: I'm struggling to quit smoking; are there any methods that can help me quit?

Section 5: Alcohol Consumption

Question 212: Does drinking alcohol cause a relapse of my disease?

Question 213: Will I feel uncomfortable after drinking alcohol?

Question 214: Do I need to limit alcohol consumption?

Question 215: Can I drink alcohol while taking medication?

Question 216: When my condition is stable, which type of alcohol is more suitable?

Question 217: Is moderate daily alcohol consumption suitable for me?

Section 6: Stress Management

Question 218: When I feel stressed, the disease seems more severe. Is stress related to inflammatory bowel disease?

Question 219: Faced with life stress, how should I adjust?

Section 7: Others

Question 220: Can I still exercise with inflammatory bowel disease?

Question 221: I often forget to take medication. Are there any methods to help me remember to take it on time?

Question 222: What should I be aware of when going out in the summer?

Chapter 9: Psychological Issues

Question 223: After getting sick, I feel very depressed. The happy me is gone. What should I do?

Question 224: I always struggle to adjust my mental state. Are there any good methods?

Question 225: When a family member is diagnosed with inflammatory bowel disease, how can I help them?

Question 226: As an inflammatory bowel disease patient, how should I face life?

Question 227: What are the benefits of establishing a support group for patients?

Chapter 10: Self-Management

Section 1: Getting Started

Question 228: I've seen many scary things about Crohn's disease online. Is it true?

Question 229: Besides asking doctors at the hospital, where can I get reliable knowledge about IBD?

Question 230: I feel scared when I see the side effects on the drug instructions. What should I do?

Question 231: I can't control myself from eating unhealthy but delicious things. What should I do?

Question 232: I am thin, but the doctor always asks me to do many blood tests. Is it bad for the body?

Question 233: Every blood test has many items. Which ones should I pay special attention to?

Question 234: Is a colonoscopy painful? What should I pay attention to?

Section 2: Advanced

Question 235: After some time of treatment, my condition has improved. What should I do next?

Question 236: There are often many arrows on the inflammatory bowel disease patient's lab report. What do they generally reflect?

Question 237: After using azathioprine, how should I monitor my blood count?

Question 238: Are there any ways to help reduce the side effects of medications and improve white blood cell and red blood cell indicators?

Question 239: I heard that symptoms and individual differences in medication for inflammatory bowel disease are quite large. How can I achieve better efficacy?

Question 240: Some people are undergoing enteral nutrition therapy with a nasal tube. Is inserting a nasogastric tube dangerous? Can I try it myself?

Question 241: I have a perianal abscess or fistula. What should I do?

Question 242: Is joining the big family of inflammatory bowel disease patients, encouraging and helping each other, important for us?

Chapter 11: Quality of Life

Section 1: General Information on Quality of Life

Question 243: When I was seeking treatment, the doctor told me that the ultimate goal of managing inflammatory bowel disease is to improve the quality of life. What exactly is the quality of life?

Question 244: Why do I need to pay special attention to the quality of life?

Question 245: If quality of life is so important, how do healthcare professionals understand my quality of life?

Section 2: Current Status and Influencing Factors of Quality of Life

Question 246: As an inflammatory bowel disease patient, what is our quality of life like?

Question 247: What factors may affect my quality of life?

Question 248: Disease activity has a significant impact on the quality of life. What ways can I use to control the condition and promote disease remission?

Question 249: Does the longer I have the disease, the worse my quality of life?

Section 3: Impact of Inflammatory Bowel Disease on Daily Life

Question 250: What possible impacts might inflammatory bowel disease have on my daily life?

Question 251: Can I still lead a normal life?

Question 252: Can I still work normally?

Question 253: Can I still study normally?

Question 254: Can I still enjoy a normal sex life?

Question 255: If I have inflammatory bowel disease, can I travel?

Section 4: Overwhelming Questions

Question 256: I'm still so young; will having inflammatory bowel disease affect my lifespan?

Question 257: Will negative emotions affect my condition?

Question 258: Should I tell others that I have inflammatory bowel disease?

Question 259: Sometimes I feel that people around me cannot understand me well. What should I do?

Question 260: I sometimes feel a lack of understanding from those around me and would like to communicate with fellow patients. What avenues are available to connect with them?

Question 261: There is some information about inflammatory bowel disease on the internet. Can I try using it?

Question 262: I have many questions about inflammatory bowel disease, but I often forget to ask during appointments. What should I do?

Chapter 12: Follow-Up

Question 263: What indicators should be checked regularly, and how often?
