# Supplementary material for: Ability of ChatGPT to Replace Doctors in Patient Education: Cross-Sectional Comparative Analysis of Inflammatory Bowel Disease
Source: J Med Internet Res. 2025 Mar 31;27:e62857. doi: 10.2196/62857 (PMC11997527; doi:10.2196/62857)
Supplement: Multimedia Appendix 2 [file jmir_v27i1e62857_app2.docx]

| 第九章  心理问题  当您得知自己或家人患IBD后，心情可能非常不好。我们非常理解您的感受。但如何面对疾病、如何调节情绪也是非常重要的大事。病友阿中、一叶、飒、650、知蓉的话也许对您有帮助。接下来第九章和第十章，听听病友们都说些什么。 | Chapter Nine  Psychological Issue  When you learn that you or your family member has been diagnosed with IBD, your mood may be very low. We truly understand your feelings. However, how to face the disease and how to regulate emotions are also very important matters. The words of fellow patients **Ah Zhong, Yi Ye, Sa, 650,** and **Zhi Rong** may be helpful to you. In the following chapters, Chapter Nine and Chapter Ten, let's hear what these fellow patients have to say. |
| --- | --- |
| 问题223：得病后，我心里非常苦闷，原来快乐的我不见了，我该怎么办？  作为您的伙伴，这问题也曾让我非常苦闷。苦闷是非常正常的心理反应，也正是因为产生了苦闷，我们才会去主动思考该怎么办？经过长期的摸索实践，我们总结出了如下经验和体会：认识它，接受它，不怕它，重视它，控制它。  一是要认识它。炎症性肠病不是癌症，但它也不是普通的肠炎，它是一种需要终身治疗的慢性肠道疾病，如同高血压、糖尿病一样，只要规范治疗就对生命没有严重的威胁，但是一旦患上这种病，必须长期治疗。目前，虽然对炎症性肠病尚无根治的办法，但只要控制得好，我们照样可以正常地工作和生活，照样可以生儿育女。它对寿命也没有什么很大的影响。  二是要接受它。我们必须要认识到“自己生病了”，而且得的是一种需要终身治疗的慢性疾病。当你学会控制它后，你会觉得其实它像高血压、糖尿病一样，没什么好怕的。只是由于炎症性肠病（特别是克罗恩病）好发于我们这些年轻人，而我们没有面对这样挫折的经验，所以难以接受它。接受这样的一个事实的确需要时间，也需要家人的理解和支持，但在我看来，最重要的还是靠自己，这是我们人生的“马拉松”。  三是要不怕它。既然定性为慢性疾病，我们就可以这样理解，不是恶性的，是有药可用的，虽然目前没有办法治愈，但可以通过药物和其他治疗手段把它控制住。这其中的过程可能是艰难的、漫长的，但你会发现，只要你积极地面对，一切都会比想象的好，因为有很多专业医生在帮助我们。尤其是当今，有比较先进的医学技术，有优质稳定的药品保障，加上亲友的抚慰和社会的关爱，我们并不孤单。因此，伙伴们大可不必惧怕，惧怕只会加剧病情的发展。如今，各种癌症患者不计其数，他们大多能从容面对，快乐地生活，其长寿者也不在少数，有的还是名人名家，他们尚能如此，我们患的又不是癌症，有什么好怕的呢？  患炎症性肠病并不可怕，可怕的是对疾病的无知，是盲目瞎整、乱折腾，不听医嘱而把简单的事情弄复杂，导致病情的恶化。曾经给我确诊的医生就微笑着跟我说：“以后就要和医院，和我们医生打一辈子的交道了。”  四是要重视它。虽然炎症性肠病不是癌症，但它也不同于常见的普通疾病，它是一种尚未被发现病因和如何治愈的疾病。我们精神上不要紧张恐惧，要尽可能放松心情，但思想上要重视它，不能麻痹大意，一旦有情况，要及时就医、果断处理。总之，我们不要嫌弃它，因为它已是我们人生的一部分，要用平常心对待它。我觉得炎症性肠病就像自家生养的一个非常调皮淘气，经常给我们惹麻烦、添烦恼的孩子，我们不能怂恿它，要用警戒之心关注它，两者并举，让它始终在你的掌控之中。  五是要控制它。在平时的生活中要细心，体察自身病情的发展变化，一旦有情况要及时与相关医生沟通联系，反馈情况，妥善处理，控制病情发展。在长期的生活实践中，我们深刻地体会到，要控制炎症性肠病，最重要的是要管理好自己。  总而言之，作为一名炎症性肠病患者，对自身所患的疾病要有一个客观正确的认识，从而勇敢地面对，并在生活实践中不断探索总结应对的方法，使自己的思想行为、精神状态、生活方式、饮食习惯、作息运动等方面始终保持在一个平稳理性的状态，确保自己的美好人生和幸福生活不受大的干扰，这才是应对疾病的根本之策。  （阿中，飒） | Question 223: After getting sick, I feel very depressed. The happy me is gone. What should I do?  As your companion, this question has also made me very depressed. Feeling down is a very normal psychological response, and it is precisely because of this sense of depression that we actively think about what to do. Through long-term exploration and practice, we have summarized the following experiences and insights: Recognize it, accept it, don't fear it, value it, control it.  First, you need to recognize it. Inflammatory Bowel Disease (IBD) is not cancer, but it is not a common gastrointestinal inflammation either. It is a chronic intestinal disease that requires lifelong treatment, similar to conditions like hypertension and diabetes. With proper treatment, it doesn't pose a severe threat to life. Although there is currently no cure for IBD, with good management, we can still lead normal lives, work, and have families. It doesn't significantly impact lifespan.  Second, accept it. We must realize that "we are sick," and it is a chronic illness that requires lifelong treatment. Once you learn to control it, you'll find that, like hypertension and diabetes, there's nothing to fear. It's just that IBD (especially Crohn's disease) tends to affect young people, and we may not have the experience of facing such setbacks, making it difficult to accept. Accepting this fact takes time, as well as understanding and support from family. However, in my opinion, the most important support comes from oneself; this is our life's "marathon."  Third, don't fear it. Since it is classified as a chronic disease, we can understand it as not malignant, there are medicines available, and although there is no cure at the moment, it can be controlled through medications and other treatment methods. The process may be challenging and lengthy, but you will find that as long as you face it positively, everything will be better than imagined because many professional doctors are helping us. Especially in today's world, with advanced medical technology, stable and quality medications, along with the comfort of friends and societal care, we are not alone. Therefore, there is no need to be afraid; fear will only exacerbate the progression of the disease. Nowadays, there are countless cancer patients, most of whom can face it calmly and live happily, some even living long lives, and some are celebrities and scholars. If they can do it, and we don't have cancer, what is there to be afraid of?  Having IBD is not frightening; what's frightening is ignorance of the disease, blindly attempting to deal with it without following medical advice, making simple matters complicated, and causing the deterioration of the condition. The doctor who diagnosed me once smiled and said, "You will be dealing with hospitals and us doctors for the rest of your life."  Fourth, value it. Although IBD is not cancer, it is different from common diseases. It is a disease with an unknown cause and cure. While we should not be nervous and fearful mentally, we should take it seriously in our thoughts. We cannot be complacent; if there is a situation, we should seek medical attention promptly and handle it decisively. In short, we should not despise it because it has become a part of our lives; we should face it with a normal mindset. I think IBD is like a mischievous and troublesome child we have raised. It often causes us trouble and adds to our worries. We should not indulge it; instead, we should pay attention to it with a vigilant heart, balancing both aspects and keeping it under control.  Fifth, control it. In daily life, be attentive and observe the development of your condition. Once there is a situation, communicate promptly with relevant doctors, provide feedback, handle it properly, and control the progression of the disease. In our long-term practical experience, we deeply understand that to control IBD, the most important thing is to manage ourselves.  In conclusion, as an IBD patient, it is essential to have an objective and correct understanding of the disease, bravely face it, continuously explore and summarize coping methods in life practice, and keep one's thoughts, behavior, mental state, lifestyle, dietary habits, and exercise habits in a stable and rational state. This ensures that one's beautiful life and happy living are not significantly disturbed, which is the fundamental strategy for dealing with the disease.  （Ah Zhong、Sa） |
| 问题224：我自己总是调整不好心理状态，有什么好办法吗？  炎症性肠病确实是个让人心烦的疾病，要是没有控制好，它就会时不时地来骚扰你，让你心神不定。我觉得伙伴们树立与炎症性肠病长期和平共处的自信心是关键，要相信自己有能力控制住它。很多伙伴一开始控制不好心态，各种情绪涌上心头，愤怒、挫折感、对未来担心等等，情绪低落，甚至丧失信心。这里，我建议伙伴们可以加入 QQ患友群或者参加一些病友间的聚会，一是那里的群友和我们同病相怜，感受相同，容易沟通，可以抱团取暖，一起树立信心；二是大家可以相互传递最新资讯达到信息共享；三是通过群友们治疗缓解的实例，可以提升自己的信心；四是通过在群里帮助新进群友可以使心情愉悦。  （阿中，一叶） | Question 224: I always struggle to adjust my mental state. Are there any good methods?  Inflammatory Bowel Disease (IBD) is indeed a bothersome condition that can disturb your peace of mind if not well-controlled. I believe that building confidence in a long-term coexistence with IBD is crucial. You need to believe in your ability to control it. Many individuals initially struggle to manage their mindset, experiencing a rush of emotions such as anger, frustration, worries about the future, and a general sense of low mood, even leading to a loss of confidence. Here, I suggest that individuals join QQ support groups or participate in gatherings with fellow patients. Firstly, the people there share the same condition, making communication easy and providing a sense of solidarity to boost confidence. Secondly, these groups facilitate the exchange of the latest information for shared knowledge. Thirdly, hearing about examples of fellow group members who have successfully managed their conditions can boost your confidence. Lastly, helping newcomers in the group can also bring joy and positivity to your own mindset.  (Ah Zhong, Yi Ye) |
| 问题225：当亲人被确诊为炎症性肠病后，我该怎样帮助他？  目前来说，炎症性肠病是需要终身治疗的，它是一个漫长的过程，只要坚持正规治疗就会取得良好的治疗效果。  生了病最怕的是无知，而教育是最好的药物，所以最好与患者一起学习炎症性肠病方面的知识。这样，作为亲人，您就可以根据患者的性格特点给予更好的引导。一要引导患者对自己有信心，调整心态，不追求完美，要有平常心，鼓励患者多与亲人朋友沟通交流，提高心理承受能力。二要引导患者养成良好的生活起居习惯，保证休息的同时也要适量运动，使患者保持有充沛的体力，提高自身抗病能力。有些看似普通的感冒对炎症性肠病患者来说可不是什么好事。三要引导患者注意合适的饮食。良好的营养是治疗炎症性肠病的关键之一。总的原则是少食多餐，保证营养的均衡。所吃的食物最好是高能量、优质蛋白质、低脂肪、低纤维少渣的。炎症性肠病患者大多缺乏叶酸，维生素A、B6、D、K，钙、铁等多种营养素，应食用富含以上营养素的食物，要忌食刺激性食物（如烟、酒及辛辣等刺激性的食物）。可以帮助患者记录引起不适反应的食物，以后可以及时避免。其实，管好炎症性肠病患者的嘴很关键，患者需要很大的勇气和毅力。作为家人，要做的不仅是提醒，更多的是引导患者有这种意识，自觉地避免这类因素。四要引导患者遵医嘱，坚持治疗。炎症性肠病的病程、症状和反应个体差异还是比较大的，包括在用药的效果上也是，不能因为某些药效果不好或自我感觉无效就放弃治疗，特别是有些药物开始起效不是那么快，但规范使用后，后期效果却是非常好。其实，炎症性肠病的治疗方法与药物是很多的，要找到一种适合患者的治疗方法，只有遵医嘱，坚持治疗，并与医生多沟通、多反馈。  （阿中） | Question 225: When a family member is diagnosed with inflammatory bowel disease, how can I help them?  Currently, IBD requires lifelong treatment, and it is a lengthy process. Good treatment results can be achieved as long as regular treatment is adhered to.  The greatest fear when one is ill is ignorance, and education is the best medicine. Therefore, it is advisable to learn about IBD together with the patient. As a family member, you can provide better guidance based on the patient's personality traits. First, guide the patient to have confidence in themselves, adjust their mindset, not pursue perfection, maintain a normal mindset, and encourage them to communicate with family and friends to improve their psychological resilience. Second, guide the patient to develop good daily habits, ensuring adequate rest while incorporating appropriate exercise to maintain physical fitness and enhance the body's ability to resist illness. Some seemingly ordinary illnesses like the common cold can be troublesome for IBD patients. Third, guide the patient to pay attention to a suitable diet. Good nutrition is crucial in treating IBD. The general principle is to eat small, balanced meals. The food consumed should be high in energy, contain quality proteins, low in fat, and low in fiber and residue. IBD patients often lack various nutrients such as folic acid, vitamins A, B6, D, K, calcium, iron, etc. They should consume foods rich in these nutrients and avoid irritating foods (such as tobacco, alcohol, and spicy foods). Helping the patient keep a record of foods causing discomfort can be beneficial for future avoidance. Managing the mouth of an IBD patient is crucial; patients need significant courage and perseverance. As a family member, what you need to do is not just remind them but guide them to have this awareness and consciously avoid such factors.  Fourth, guide the patient to follow medical advice and adhere to treatment. The course, symptoms, and individual differences in the response to IBD vary significantly, including the efficacy of medications. Patients should not give up treatment simply because certain medications seem ineffective or self-perceived as such. Some medications may take time to show their effects, but with proper use, the later effects can be very positive. In fact, there are many treatment methods and medications for IBD. Finding a treatment method that suits the patient is crucial. Only by following medical advice, adhering to treatment, and communicating with the doctor can the best results be achieved.  (Ah Zhong) |
| 问题226：作为一名炎症性肠病患者，我要怎么去面对生活？  在病友之间，大家非常喜欢一句话，那就是“精彩生活永相伴”。我们和正常人一样，有着美好生活的权利和能力。我们的目标是“与病和谐共处，带病精彩生活”。  但由于疾病，我们在生活中会遇到许多的问题，从最基本的吃到工作再到结婚生儿育女，都需要我们用勇气和智慧去面对，且会比正常人难一些，因为 IBD本身就是一个很大的挑战。但在我看来，如果伙伴们连 IBD都可以搞定，那么其他事情更是“小菜一碟”了。  生活中，我们遇到最多的尴尬就是饮食方面的问题了，经常会有亲朋好友约你，而我们在饮食方面会有较多的忌口。所以我的建议是可以跟比较亲近的人用“肠胃不太好”这样的字眼来形容自己的身体状态，以避免摄入会引起不适的食物。在身体允许的情况下，尽可能地融入社交圈，不要在心理上把自己归为“异类”而自我封闭。  在学习、工作、生活方面，也要保持积极乐观向上的心态，把自己当一个健康人，在自己的领域内正常运作。同时要避免过度操劳、心情起伏。  由于疾病本身的原因，有时我们非常容易陷入悲观的负面情绪，或是对手术的恐惧，或是对人生没信心等，而这种负面的情绪会加重我们的病情。我的建议是可以在低落阶段多看看励志的书籍，比如我个人比较喜欢看的有史铁生的《我与地坛》、于娟的《此生未完成》及凌志军的《重生手记》。他们都有一个共同点，在生病后，顽强与疾病共处。  最后送大家一句话：记住自己是一个患者，然后像一个健康人一样去享受生活！  （阿中，飒） | Question 226: As an inflammatory bowel disease patient, how should I face life?  Among fellow patients, there is a favorite saying, "A splendid life always accompanies us." Like everyone else, we have the right and ability to lead a beautiful life. Our goal is to "harmoniously coexist with the disease and lead a splendid life despite it."  However, due to the illness, we encounter many challenges in life, from basic necessities like eating to work and even getting married and raising children. All of these require us to face them with courage and wisdom, and it might be a bit more difficult than for the average person because IBD itself is a significant challenge. But in my view, if fellow patients can manage IBD, then other things are just a "walk in the park."  In daily life, one of the most common awkward situations we face is related to dietary issues. Often, friends and family may invite us, and we have specific dietary restrictions. My suggestion is to use phrases like "my digestive system is not very good" when describing your health condition to close people, to avoid consuming foods that may cause discomfort. When possible, try to integrate into social circles, avoiding categorizing yourself as an "outsider" and self-isolating psychologically.  In terms of learning, work, and daily life, maintain a positive, optimistic, and upward mindset. Treat yourself as a healthy individual and operate normally within your field. At the same time, avoid overexertion and mood swings.  Due to the nature of the disease, we may easily fall into pessimistic negative emotions, whether it's fear of surgery or a lack of confidence in life. Such negative emotions can exacerbate our condition. My suggestion is to read inspirational books during low periods. Personally, I enjoy reading books like "Myself and Ditan" by Shi Tiesheng, "This Life is Unfinished" by Yu Juan, and "Rebirth Memoirs" by Ling Zhijun. They all share a common point of perseverance in facing illness.  In conclusion, remember that you are a patient, and then enjoy life as if you were a healthy person!  (Ah Zhong, Sa) |
| 问题227：建立患友俱乐部的意义有哪些呢？  绝大多数的炎症性肠病患者是年轻人，对我们来说，绚丽的人生才刚刚开始，我们在父母的羽翼下长大，根本谈不上有什么人生阅历，一下子让我们面对这样的现实，十有八九会被打趴下，一蹶不振。而患友俱乐部这个平台可以将我们这些年轻患者聚集在一起，大家一起积极地面对炎症性肠病，充分认识和了解炎症性肠病，积极配合医生治疗，积极管理自己，进而增强自我照顾的能力，获得心理上的支持。在现实的实践中，我们发现通过患友俱乐部可以实现一些医生做不到的事情。患友俱乐部既可以是一种很好的宣教方式，又可以是一种很好的心理引导方式，会潜移默化地教会患者如何面对疾病并正常生活。  建立俱乐部还有着更深远的意义，通过向社会呼吁，引起社会的关注，逐渐获得社会对患者群体的支持，改善患者的生活现状，包括我们的社会保障、医疗保障等。从社会学的角度来看，关注到炎症性肠病患者，就是关注到您自家的孩子，因为炎症性肠病的发病率在我国正以10倍的速度逐年上升。除此之外，成立患友组织，实际上还可以推动对这个疾病的研究，这是我们患者最深切的一个期望，希望加快对炎症性肠病的研究，并且能够在攻克这个疾病方面有新的重大突破，以造福我们广大患者。  （阿中） | Question 227: What are the benefits of establishing a support group for patients?  The majority of IBD patients are young individuals, and for us, life is just beginning. Growing up under the wings of our parents, we lack significant life experiences. Suddenly facing the reality of a chronic illness, we are likely to be knocked down and find it challenging to recover. A support group provides a platform to gather young patients together, encouraging proactive engagement with IBD, fostering a deep understanding of the condition, promoting active cooperation with medical treatments, and enhancing self-management skills. In practical terms, we've observed that a support group can achieve things that individual doctors might find challenging. It serves not only as an excellent educational tool but also as a means of psychological guidance, subtly teaching patients how to cope with the disease and lead a normal life.  Establishing a support group holds broader significance. By raising awareness and garnering societal attention, gradual support can be obtained for the patient community, improving the living conditions of patients, including aspects such as social security and medical coverage. From a sociological perspective, showing concern for IBD patients is tantamount to showing concern for your own children, given the alarming increase in the incidence rate of IBD in our country at tenfold speed annually. Additionally, founding a patient support organization can effectively propel research on this disease. This aligns with the deep-seated hope of patients for accelerated research on IBD, anticipating groundbreaking progress in overcoming this illness to benefit the wider patient community.  (Ah Zhong) |
| 第十章  自我管理 | Chapter Ten  Self-management |
| 一、入门篇 | One: Getting Started |
| 问题228：在网上看到许多关于克罗恩病的说法，有的说得非常可怕，是真的吗？  首先，你可以放心，克罗恩病不是癌症，它是一种慢性的消化道炎症，只是因为开始发病时往往不易被发现，所以容易错过治疗，时间久了就会造成并发症，而这些并发症如果严重的话，确实是会对生命带来威胁，或是对日后的生活产生很大的影响。  所以只要控制好克罗恩病，坚持治疗，就可以减少并发症的发生，和正常人一样生活。并且可以告诉你的是，根据数据研究，我们的生存寿命不会因为得了 IBD而缩短，而是和正常人基本一样。当然，极少数患者因为严重并发症是会影响到寿命的。  （阿中） | Question 228: I've seen many scary things about Crohn's disease online. Is it true?  Firstly, you can rest assured that Crohn's disease is not cancer. It is a chronic inflammatory condition of the digestive tract. The challenge often lies in its early detection, making it easy to overlook for treatment. Over time, this can lead to complications, which, if severe, can indeed pose a threat to life or significantly impact future quality of life.  Therefore, as long as Crohn's disease is well managed and treatment is adhered to, the occurrence of complications can be minimized, allowing individuals to lead a life similar to that of a person without the condition. Additionally, based on research data, our life expectancy is not significantly shortened due to inflammatory bowel disease (IBD); it is generally comparable to that of the general population. Of course, a very small percentage of patients may experience a shortened lifespan due to severe complications.  (Ah Zhong) |
| 问题229：除了去医院问医生，我还可以从哪里了解到可靠的IBD知识？  如今网络非常发达，但网络上的信息有时却是真假难分，你可以通过一些网络上的专业期刊找到你想要的一些知识，比如通过万方数据资源、 CNKI数字图书馆、维普资讯资源等搜索你想了解的关键词。你还可以通过手机微信关注与 IBD相关的公众号或订阅号（向您推荐我们的“爱在延长”微信订阅号），里面会定期发布与 IBD相关的信息。当然，你也可以去书店购买相关书籍学习（比如现在您在看的这本书）。  （阿中） | Question 229: Besides consulting doctors at the hospital, where else can I obtain reliable information about IBD?  In today's era of advanced technology, the internet is a valuable resource. However, it can be challenging to distinguish between accurate and misleading information online. You can explore reliable knowledge through professional journals on the internet, such as using databases like Wanfang Data, CNKI Digital Library, and VIP Information Resources. Another avenue is to follow WeChat public accounts or subscription accounts related to IBD on your mobile phone (we recommend our "Love in Prolongation" WeChat subscription account), where regular updates about IBD are provided. Of course, you can also purchase relevant books from bookstores for in-depth learning (like the one you are currently reading).  (Ah Zhong) |
| 问题230：看到药品说明书上的副作用，我感觉很害怕，怎么办？  俗话说“是药三分毒”，但也可能就是因那三分毒对我们的疾病有治疗效果，比如硫唑嘌呤这种免疫抑制剂就是通过减少体内白细胞来达到控制病情的效果的。所以我们要根据疾病的情况来具体客观地分析，用药对我们来说是利大还是弊大，只要把握好这个原则，治疗的方向就不会有太大偏差。同时还可以寻找一些方法减少药物的副作用对我们的影响。最重要的是你需要找到专业的医师，和医师一起讨论合适的治疗方法，监测药物的副作用。  （阿中） | Question 230: I feel scared when I see the side effects on the drug instructions. What should I do?  As the saying goes, "Every medicine has three parts poison," but it's also those three parts that may have a therapeutic effect on our disease. For example, immunosuppressive drugs like azathioprine achieve disease control by reducing the number of white blood cells in the body. Therefore, it's crucial to objectively analyze the specific situation of the disease and determine whether the benefits outweigh the risks when it comes to medication. By adhering to this principle, the treatment direction is less likely to deviate significantly. Additionally, you can explore methods to minimize the impact of drug side effects. Most importantly, you need to consult with a professional physician, discuss appropriate treatment methods, and monitor the side effects of medications.  (Ah Zhong) |
| 问题231：看到那些虽然不健康但非常好吃的东西，总是控制不住去吃，怎么办？  炎症性肠病发病位置是消化道，饮食将直接影响我们的疾病，所以保证食品的卫生和健康对我们来说意义重大。当然，如果在病情控制缓解的情况下，同时保证食品卫生，偶尔少量地吃一些类似炸鸡之类的不健康的食品，可以让我们心情愉悦，亦无大碍，但是一定要把握好度。特别是在病情不稳定的时候，切不可任性，否则最终伤的还是自己。我更希望的是通过你的努力自然地保持良好的养生习惯，戒烟戒酒，少食多餐，早睡早起，适当锻炼，保持心态，那才是真正的棒！  （阿中） | Question 231: I can't control myself from eating unhealthy but delicious things. What should I do?  Inflammatory bowel disease affects the digestive tract, and dietary choices directly impact our condition. Ensuring food hygiene and health is of great significance for us. Of course, if the disease is under control and food hygiene is maintained, occasionally indulging in small amounts of unhealthy foods, such as fried chicken, for the sake of enjoyment is acceptable. However, it's crucial to exercise moderation. Especially during periods of disease instability, it's important not to indulge recklessly, as it could ultimately harm your health. I encourage you to naturally maintain good dietary habits through your efforts—quit smoking and drinking, eat small, frequent meals, get enough sleep, exercise appropriately, and maintain a positive mindset. That's the real key to well-being!  (Ah Zhong) |
| 问题232：我很瘦，但医生总让我验许多的血液项目，这会对身体不好吗？  在病情不太好的时候，我们确实是要经常地去化验许多的血液项目，因为相对于做肠镜或 CT来说，这样的检查对我们身体的损伤是非常微小的，同时也可以直接反映病情的趋势和严重程度，对治疗方案有着极为重要的参考性，所以牺牲这一点点血液还是很有必要的。你不必担心每次抽好几管血会影响你的身体，因为我们人体的血液是在不断更新的，检查需要的血液不会很多，最多10～20毫升，几乎对身体没有影响，这和献血可不是一回事。  （阿中） | Question 232: I am thin, but the doctor always asks me to do many blood tests. Is it bad for the body?  When the condition is not very good, it is indeed necessary for us to undergo frequent blood tests. Compared to procedures like colonoscopies or CT scans, these blood tests cause minimal damage to our bodies. They can directly reflect the trend and severity of the disease, providing crucial reference points for treatment plans. Sacrificing a small amount of blood for these tests is necessary. You don't need to worry that drawing several tubes of blood each time will affect your body. Our body's blood is constantly renewing, and the amount of blood needed for these tests is minimal—usually 10 to 20 milliliters. This has almost no impact on the body and is quite different from blood donation.  (Ah Zhong) |
| 问题233：每次的化验单上都有很多项目，哪些是我要特别关注的？  我们 IBD患者要特别关注的指标有以下这些。  （1）血液项目有：血常规中的白细胞、 CRP（ C反应蛋白）、ESR（血沉）、中性粒细胞、红细胞计数、血小板计数；血生化中的总蛋白、谷丙转氨酶、肌酐。  （2）粪便项目有：粪便常规中的隐血、白细胞、寄生虫；粪便钙卫蛋白的检测结果可以直接反映 IBD的炎症程度。  （3）尿常规中有：红细胞、尿蛋白、胆红素。  （650，阿中） | Question 233: Every blood test has many items. Which ones should I pay special attention to?  IBD patients should pay special attention to the following indicators:  (1) Blood tests include: White blood cells, CRP (C-reactive protein), ESR (erythrocyte sedimentation rate), neutrophils, red blood cell count, platelet count in a complete blood count; total protein, alanine transaminase, and creatinine in blood biochemistry.  (2) Stool tests include: Occult blood, white blood cells, parasites in routine stool tests; the results of fecal calprotectin testing can directly reflect the degree of inflammation in IBD.  (3) Urine tests include: Red blood cells, urinary protein, and bilirubin in a routine urinalysis.  (650, Ah Zhong) |
| 问题234：做肠镜会不会很痛？我要注意些什么？  现在做肠镜有两种方法，一种是普通的，另一种是无痛的。前者多少会有些不适；后者是在全麻下进行的，所以不会有任何不适，只是会贵些。每个人的肠道情况不一样，医师技术水平也不同，因此不适的情况因人而异。我做过7次肠镜，都是普通的那种，而且现在的肠镜机器都很高级，医生的技术也非常精湛，只要掌握了正确的操作方法，肠镜检查可以做到无痛，只会有些不适而已，你完全没必要因为恐惧心理而主动放弃肠镜检查。  肠镜前的准备主要是肠道清洁，而清肠的方法也有很多种，具体的在做检查之前，医生或护士会发给你一张告知单，上面会告诉你安排肠镜的时间和清肠药物的使用方法。就我的经验来说，我们只要注意这几个方面就可以了。①可以提前2天就开始肠道准备，饮食清淡，吃些好消化、残渣少的食物，如豆腐和鱼等。②服用清肠药物一定要按说明来，直到排出水样便（呈清水或淡黄色，无粪渣），这很关键。③由于清肠和饮食的要求，过程中可能有低血糖的反应，你可以备几颗糖果救急。④在检查时放松心情，不要过于紧张，因为你放松了，肠子才会松弛，肠镜的异物反应也会减少，从而减少不适感。⑤检查过程中，为了便于进镜或看清肠腔的黏膜形态，医生有时需要向肠腔内注入少量空气以扩张或暴露肠腔，此时患者常感到腹胀，有想解大便的感觉，这很正常。当你感觉非常不适的时候，你可以做做深呼吸，让自己尽量地放松。⑥由于大肠总长度为1.5～2.0米，在腹腔里弯曲迂回且不固定，所以你要尽可能地配合医生做好翻身屏气等动作，以顺利地完成检查。⑦检查完后可能还会有些不适，主要表现为腹胀，那是因为肠镜检查过程中注入了一些空气，等屁放出后，不适感就会消失，不必恐慌。⑧肠镜后的饮食还是要慢慢来，千万别马上就大餐一顿，那可能让你更难过。  （阿中） | Question 234: Is a colonoscopy painful? What should I pay attention to?  Currently, there are two methods for colonoscopy: a regular one and a painless one. The regular method may cause some discomfort, while the painless method is performed under general anesthesia, ensuring there is no discomfort, but it may be more expensive. The level of discomfort varies from person to person, depending on individual bowel conditions and the physician's skill. I have undergone colonoscopy seven times, all using the regular method. With advanced colonoscopy machines and skilled doctors, the procedure can be virtually painless, causing only mild discomfort. There is no need to voluntarily forgo colonoscopy due to fear.  The preparation for a colonoscopy mainly involves cleansing the bowel, and there are various methods for bowel cleansing. Before the examination, the doctor or nurse will provide you with an instruction sheet detailing the colonoscopy schedule and how to use bowel-cleansing medications. Based on my experience, you should pay attention to the following aspects:  Start preparing the bowel two days in advance with a light diet, including easily digestible and low-residue foods such as tofu and fish.  Follow the instructions for taking bowel-cleansing medications until producing watery stools (clear or light yellow, without fecal residue). This is crucial.  Due to the dietary and bowel-cleansing requirements, you may experience symptoms of low blood sugar. Have some candies on hand for emergency relief.  Relax during the examination; don't be overly nervous. Relaxing helps the intestines to loosen, reduces the colonoscope's reaction, and minimizes discomfort.  During the examination, the doctor may inject a small amount of air into the intestinal cavity to expand or expose the mucosal morphology, which can cause a feeling of abdominal distension or an urge to defecate. This is normal. When you feel very uncomfortable, take deep breaths to relax.  Due to the large and flexible nature of the colon (1.5 to 2.0 meters in total length), cooperate with the doctor in turning over and holding your breath to facilitate a smooth examination.  After the examination, you may experience some discomfort, mainly bloating. This is because some air was injected during the colonoscopy. Once the gas is expelled, the discomfort will disappear, so there is no need to panic.  Be cautious with your diet after the colonoscopy; don't indulge in a big meal immediately, as it may make you feel worse.  (Ah Zhong) |
| 二、进阶篇 | Two: Advanced Level |
| 问题235：在治疗一段时间后病情有所缓解，下一步我要怎么办？  当我们的病情得以控制，有所缓解时，千万不要掉以轻心，因为它还在那，只是在我们的努力下它变得老实了，所以还要继续维持治疗。当然，在这段时间里也要与你的主治医生保持联系，可以和他们商量你以后的计划，比如是不是可以外出旅行，计划生个健康的宝宝，对纤维化的狭窄进行择期手术，以及缓解期的药物调整等。  （阿中，650） | Question 235: After some time of treatment, my condition has improved. What should I do next?  When your condition is under control and has improved to some extent, it's crucial not to become complacent. The disease is still there; it's just behaving better due to your efforts. Therefore, it's essential to continue with the treatment. During this period, maintain regular contact with your primary doctor. Discuss your future plans with them, such as the possibility of traveling, planning for a healthy pregnancy, scheduling surgery for fibrotic strictures, and adjusting medications during the remission phase.  (Ah Zhong; 650) |
| 问题236：炎症性肠病患者的化验单上时常会有很多箭头，它一般反映什么？  1.血液项目  （1）白细胞（WBC）：也俗称白血球，是我们人体抵抗细菌入侵的屏障。如果白细胞增高了，说明 IBD炎症高了；如果过低了，又会让我们的身体容易被细菌侵入。所以我们在服用美沙拉嗪和免疫抑制剂时要监测这个指标。  （2） C反应蛋白（CRP）：在身体受到感染或组织损伤时，CRP会上升得特别快。还有一种叫超敏C反应蛋白（hs-CRP）。它们都是监测 IBD炎症状态的灵敏指标，当该指标过高时，应该遵医嘱使用抗生素治疗。  （3）血沉（ESR）：对于 IBD患者来说，血沉加速（值高），表示病情复发和活跃；当病情缓解时，血沉也逐渐恢复到正常值。有病友根据自己的情况总结说血沉主要体现肠道炎症的范围，而 CRP主要体现肠道炎症活动的程度。  （4）中性粒细胞（GRAN）：前面说白细胞是我们人体抵抗细菌入侵的屏障，其实在这个屏障中起到最大作用的是中性粒细胞，它是白细胞中的一种，占白细胞总数的50%～70%。当我们人体受感染时，其比例会显著增高，但中性粒细胞在消灭细菌后，自身也常坏死，所以中性粒细胞过低比白细胞过低更值得我们关注。  （5）血红蛋白（HGB）：主要反映我们是否有贫血。IBD患者由于肠道有炎症，一方面，肠道吸收不好会造成贫血；另一方面，也可能因肠道出血引起贫血。这时，可以配合化验大便的潜血情况来判断是不是肠道出血造成贫血，如出血严重必须尽快急诊。  （6）血小板（PLT）：它的高低也是反映 IBD患者肠道出血情况和炎症情况的一种。硫唑嘌呤和美沙拉嗪类药物也会造成血小板减少。  （7）谷丙转氨酶：这是与肝功能有关的一个重要指标。由于 IBD患者吃的很多药物的成分需要通过肝脏代谢，所以有时会引起肝脏功能的损伤。由于我们每个人的体质不同，所以肝脏对药量的耐受也不一样。因此，我们要定期监控。一般情况下，药物引起的肝脏功能损伤在停药后就会恢复正常。  （8）血肌酐：它是一个与肾功能相关的重要指标。大家都知道，肾脏是尿液生成的地方，它可以把血液中不好的一些物质通过肾小球的过滤排出体外。而 IBD患者吃的药物通过胃肠道吸收后都会进入血液中，由于这些成分会对肾脏功能有所影响，当肾小球滤过率下降到正常的50%以上时，血肌酐就开始迅速上升，因此当血肌酐明显高于正常值时，常表示肾功能已严重受损，所以我们要加以监控。  2.粪便项目  （1）粪便常规：正常的粪便应为黄色段状软便，但有时也会受饮食、炎症等因素的影响产生一些形状及颜色的变化（如有些肠内营养制剂会造成粪便成深绿色），我们光凭肉眼并不能判断，这就需要一些专业的手段来检测大便情况是否正常，比如简单又方便的大便常规检测。这其中的隐血检测项目可以检查出大便中极少量甚至肉眼看不见的出血，化验单上“＋”出现的个数表示出血情况的严重程度。“＋”越多，出血情况越严重。此外，通过检测粪便的白细胞、寄生虫等，能了解消化道是否存在细菌感染、寄生虫感染等情况。  （2）粪便钙卫蛋白：是用于鉴别 IBS（肠易激综合征）与 IBD的一项检测手段，也是检测 IBD活动性的一项指标。由于标本直接来源于肠道，所以它的检测价值优于 CRP、血沉（ESR），且与肠镜检测有很好的相关性，能客观地反映肠道炎症情况。对 IBD患者来说，钙卫蛋白指数较高，则提示病情活动；当钙卫蛋白指数较低时，则提示病情较为稳定。  3.尿项目  尿常规：可以反映一些肾脏病变以及身体其他脏器影响尿液改变的疾病，如糖尿病、肝胆疾病等。IBD患者在服用柳氮磺吡啶、免疫抑制剂等药物时，定期监控尿常规可以提早发现药物对身体产生的副作用。当尿常规里出现红细胞、尿蛋白或胆红素偏高时，要及时去医院就诊。  （阿中，知蓉） | Question 236: There are often many arrows on the inflammatory bowel disease patient's lab report. What do they generally reflect?  Blood Tests  (1) White Blood Cells (WBC): Commonly known as white blood cells, they act as a barrier against bacterial invasion. An elevated WBC count indicates increased inflammation in IBD. A low count can make the body susceptible to bacterial invasion. Monitoring this indicator is crucial when taking medications like mesalazine and immunosuppressants.  (2) C-Reactive Protein (CRP): CRP rises rapidly in the body in response to infection or tissue damage. There is also a highly sensitive form called high-sensitivity C-reactive protein (hs-CRP). Both are sensitive indicators of IBD inflammation. Elevated levels may warrant antibiotic treatment as per medical advice.  (3) Erythrocyte Sedimentation Rate (ESR): In IBD patients, an accelerated ESR (high value) indicates disease recurrence and activity. As the condition improves, ESR gradually returns to normal. Some patients believe ESR mainly reflects the extent of intestinal inflammation, while CRP mainly indicates the degree of inflammation activity.  (4) Granulocytes (GRAN): These play a significant role in the immune response, with neutrophils being crucial. An increase in their proportion signifies infection. Neutrophil levels lower than normal are more concerning than low white blood cell counts.  (5) Hemoglobin (HGB): Reflects the presence of anemia. IBD patients may experience anemia due to poor absorption in the inflamed intestines or bleeding. Assessing fecal occult blood, in conjunction with HGB levels, helps determine if anemia is due to intestinal bleeding, requiring urgent attention in severe cases.  (6) Platelets (PLT): Platelet levels indicate IBD patients' intestinal bleeding and inflammation. Medications like azathioprine and mesalazine can lead to a decrease in platelet count.  (7) Alanine Aminotransferase (ALT): An important indicator related to liver function. Since many medications taken by IBD patients are metabolized in the liver, liver function impairment may occur. Regular monitoring is essential as individuals tolerate medication doses differently.  (8) Serum Creatinine: An important indicator related to kidney function. As medications absorbed in the gastrointestinal tract enter the bloodstream, they may affect kidney function. Significant elevation in serum creatinine suggests severe kidney damage, requiring close monitoring.  Stool Tests  (1) Stool Routine: Detects abnormalities in stool color and shape, which may be influenced by diet, inflammation, etc. Hidden blood testing can reveal even microscopic bleeding, with the number of "+" signs indicating the severity. Additionally, detecting white blood cells and parasites helps assess bacterial or parasitic infections in the digestive tract.  (2) Fecal Calprotectin: Used to differentiate between Irritable Bowel Syndrome (IBS) and IBD, serving as an indicator of IBD activity. As it directly originates from the intestines, its value is superior to CRP and ESR, showing good correlation with colonoscopy results. Higher calprotectin levels suggest active disease, while lower levels indicate a more stable condition.  Urine Tests  Urine Routine: Reflects kidney abnormalities and other diseases affecting urine due to various organ influences, such as diabetes, liver, and gallbladder diseases. Regular monitoring of urine routine while taking medications like sulfasalazine and immunosuppressants can help detect potential side effects early. Timely medical attention is needed if red blood cells, protein, or bilirubin levels are elevated in the urine. |
| 问题237：使用硫唑嘌呤治疗后，我要怎么样监控我的血象？  炎症性肠病是由于免疫系统过度活跃造成消化道炎症所致的，所以我们可能使用抑制免疫系统的药物帮助控制疾病。比如硫唑嘌呤就是常用的一种免疫抑制剂，它通过减少体内白细胞来作用于疾病，由于起效需要大约3个月的时间，所以一般要维持原来的药物或者加量直到免疫抑制剂开始起效，再把原来的药物逐渐减完。平时常与激素配合使用，硫唑嘌呤一般从小剂量开始使用：使用后的第一周，您需要验一次血常规；第二周验一次血常规和肝肾功能；第三周、第四周，分别和第一周、第二周一样。这样的血液检查非常重要，硫唑嘌呤是通过减少体内白细胞来控制病情的，如果白细胞太低就会有感染的风险，一般情况在3.5×109/L（升）以上是相对安全的；硫唑嘌呤通过肝脏分解代谢，故监测肝功能的血液检查也非常重要，可与肾功能和其他血液检查同时进行。然后慢慢增加药量，逐步达到适合你的有效剂量，中间可能还需要根据你的验血情况进行多次调整。当你的药物剂量稳定，血液检查正常时，就可以拉长验血的周期，具体验血时间请遵医嘱。  （阿中） | Question 237: After using azathioprine, how should I monitor my blood count?  Inflammatory bowel disease (IBD) is caused by overactivity of the immune system leading to inflammation in the digestive tract. Therefore, immunosuppressive drugs may be used to help control the disease. Azathioprine is a commonly used immunosuppressant that works by reducing white blood cells in the body. Since it takes about three months to take effect, the usual practice is to maintain the original medication or increase the dosage until the immunosuppressant starts working, and then gradually taper off the initial medication. It is often used in conjunction with steroids.  Azathioprine is typically initiated at a low dosage. During the first week of use, you should have a blood test. In the second week, both a blood test and liver and kidney function tests are recommended. The third and fourth weeks follow the same testing schedule as the first and second weeks. These blood tests are crucial because azathioprine controls the disease by reducing white blood cells. If the white blood cell count is too low, there is a risk of infection. Generally, it is considered relatively safe when the white blood cell count is above 3.5×10^9/L (per liter). Since azathioprine is metabolized in the liver, monitoring liver function through blood tests is also essential and can be done simultaneously with kidney function and other blood tests.  Subsequently, the medication dosage should be gradually increased to reach an effective level tailored to your condition. Adjustments may be necessary based on your blood test results. Once the medication dosage stabilizes and blood tests show normal results, the frequency of blood tests can be reduced, following your doctor's advice.  (Ah Zhong) |
| 问题238：有什么办法可以帮助减少药物的副作用，让白细胞、红细胞指标好一点？  如前所述，硫唑嘌呤是通过减少体内白细胞来控制病情的，而激素是会让白细胞上升的药物，当两者配合使用时，一般是激素慢慢减量，硫唑嘌呤慢慢增量，当激素减到一定程度时，白细胞就会下降得特别快，这时候我们就可以用一些副作用极小的药物或没有副作用的食物来帮助我们提高白细胞、红细胞值。药物方面，医生比较有经验，您可以遵医嘱。食物方面，可以用“五红水”（即取15～30克的红枣、红小豆、枸杞、红皮花生，加水煮开后，再加入适量红糖即可），一天喝一次。还有牛尾汤也可以有利于白细胞生长，平时还可以喝点绿豆汤排毒。除了食品，我们还可以拍打胆经促进排毒，捏手指上小太阳下第一个关节处也可以刺激白细胞的生长。适当的体育锻炼对我们的白细胞生长也有利。以上这些办法的关键是要坚持，不是一两次就能见效，当然最关键的还是加强平时的营养。  （阿中，650） | Question 238: Are there any ways to help reduce the side effects of medications and improve white blood cell and red blood cell indicators?  As mentioned earlier, azathioprine controls the disease by reducing white blood cells, while steroids are drugs that can increase white blood cell count. When used in combination, the general approach is to gradually taper off steroids while slowly increasing azathioprine. When steroids are reduced to a certain extent, white blood cell count can drop rapidly. At this point, we can use medications with minimal side effects or incorporate foods without side effects to help increase white blood cell and red blood cell counts.  In terms of medications, it's advisable to follow your doctor's experienced guidance. For dietary considerations, a concoction known as "Five Red Soup" can be prepared (comprising 15–30 grams each of red dates, adzuki beans, goji berries, and red-skinned peanuts, boiled with water, and then adding an appropriate amount of brown sugar). This soup can be consumed once a day. Additionally, oxtail soup is believed to be beneficial for white blood cell growth, and regularly drinking mung bean soup can aid in detoxification. Besides food, tapping the gallbladder meridian to promote detoxification and applying pressure to the first joint below the small finger's nail (little sun point) can stimulate white blood cell growth. Moderate physical exercise is also favorable for white blood cell growth. The key to these methods is consistency; they are not a one-time solution. Of course, the most crucial aspect is to enhance regular nutrition.  （Ah Zhong；650） |
| 问题239：听说炎症性肠病的症状和用药的个体差异都比较大，我要怎样才能获得更好的疗效？  主观方面我们要做的：炎症性肠病的治疗是一个长期的过程，很可能是长伴一生的。在这么长的时间里，光被动地接受医生的治疗是远远不够的（毕竟一次就诊最多十几分钟，医生也不可能个个进行追踪随访）。所以自我管理就显得尤为重要，当然自我管理并不意味着我们就不需要医生指导，自己摸着石头过河。恰恰相反，我所理解的自我管理是“在医生的保驾护航下，自己照顾自己”。让自己从一个被动的接受者，转变为治疗过程中积极的参与者。  客观方面我们要做的：  （1）病情发展要心中有数，做好病历的整理，包括以往的肠镜、CT、磁共振（MRI）、血液等检查报告，特别是老患者建议最好有自己的病情概述当首页。如果可能的话，最好将 CRP、血沉、白细胞等关键检查指标的历次数据汇总在一张表上，这样方便医生和自己更快、更好地了解病情发展情况。其实，类似的记录还有很多，如记录日常的体温、体重、大便的次数及形态、不适的情况与程度、药物的吃法与剂量。  （2）按时服药，定期医学检查，改变膳食。可以做饮食日记，跟踪自己的饮食习惯，把每天的饮食情况记录下来，对照前一条看看有无相关性，筛选出易引起自己肠胃不适的食品。可以在食用某食品或饮料后，在表格上记录下相应的名称，然后隔段时间记录下是否有腹痛、腹泻、腹胀等症状。坚持一个月以后，可以基本得出哪些食物或饮品不耐受，把它们剔除出自己的食品清单。  （3）做到生活有常，劳逸结合，量力而行，建立和保持在工作、家庭、朋友中的新角色。缓解期的炎症性肠病患者完全可以正常地工作、学习、生活，而正常的社交活动也可以给患者带来正面的积极的影响。  （4）还可记录在每次发生情况时，医生是如何处理的，久而久之就能总结出适合你的治疗方法和规律。  （阿中，一叶） | Question 239: I heard that symptoms and individual differences in medication for inflammatory bowel disease are quite large. How can I achieve better efficacy?  On the subjective aspect, what we need to do is self-management. The treatment of IBD is a long-term process, possibly a lifelong companion. In such a long time frame, merely passively accepting the doctor's treatment is far from enough (considering a doctor's visit lasts at most fifteen minutes, and it's not possible for doctors to follow up individually). Hence, self-management becomes crucial. Self-management doesn't mean we don't need guidance from the doctor; it means taking an active role in caring for oneself under the doctor's guidance. It's about transforming from a passive recipient to an active participant in the treatment process.  On the objective aspect, here's what we need to do:  Be aware of the progression of the disease and organize your medical history, including past reports from colonoscopies, CT scans, magnetic resonance imaging (MRI), blood tests, etc. For older patients, it is advisable to have a summary of their medical condition on the first page. If possible, it's best to consolidate the historical data of key indicators like CRP, ESR, and white blood cells on a single table. This makes it easier for both the doctor and yourself to understand the development of the disease quickly and effectively. In fact, similar records can be kept for various aspects, such as recording daily body temperature, weight, frequency and form of bowel movements, discomfort levels, and details of medication usage.  Take medication on time, undergo regular medical check-ups, and modify your diet. Keeping a food diary to track dietary habits is useful. Record your daily food intake, and correlate it with the previous entry to check for correlations. Note down the name of the food or beverage consumed and, after some time, record whether there were symptoms like abdominal pain, diarrhea, bloating, etc. After maintaining this for about a month, you can identify which foods or drinks you might be intolerant to and eliminate them from your diet.  Maintain a regular lifestyle, balance work and rest, and establish and maintain new roles in work, family, and social circles. Patients with IBD in remission can lead a normal life in terms of work, study, and daily activities. Engaging in normal social activities can have positive effects on patients.  Record how doctors handle each situation when it occurs. Over time, you can summarize the treatment methods and patterns that suit you.  （Ah Zhong；Yi Ye） |
| 问题240：有的伙伴在鼻饲治疗，插鼻胃管有危险吗？我可以自己尝试吗？  其实鼻饲没有想象的那么可怕，更多的是一种心理上的抗拒，当你在心理上接受它了，那么在执行上就会非常自然。因为与口服肠内营养比较，鼻饲肠内营养在执行力和对肠胃负担上要有更多的优势。一般情况下，插鼻胃管是由医生执行的。但在实践中我们发现，自己插比医生插得更顺利，因为自己可以感觉胃管所处的角度和位置，自己可以把握插入的时机并更好地做一些配合的吞咽动作。一般情况下，清醒状态下的人是不可能插到气管而没有反应的，所以完全可以自己尝试插胃管，而且应该鼓励自己插胃管。对那些很多药物都不能耐受的伙伴来说，这可能就是一种控制疾病的治疗方法。  具体插鼻胃管的方法如下。  1.准备工作  （1）先将手洗干净，确保卫生这一条件很重要。  （2）备好鼻胃管、针筒、记号笔、纸胶布（3M的比较好，不过敏）、剪刀及一杯水。  （3）插管前可以试下两边鼻孔哪边比较通畅，以确定插哪个鼻孔。  （4）测量自己鼻胃管需要插入的长度。先将鼻胃管的头部（非接口的一端）从发际测至剑突（胸骨中间凹陷的软处）或从鼻尖测至耳垂再测至剑突，测量过程中请站直。测好后请记住鼻胃管上的刻度，如果没有，可以用笔或胶布做下记号，后面插管时基本就插到这个长度。  2.插管步骤  （1）先喝一口水润滑下口腔和食管。  （2）让鼻胃管头朝下自然下垂，找到头端自然弯曲的方向。  （3）手拿在鼻胃管头端10厘米处，让自然弯曲的方向朝向自己。  （4）手持鼻胃管指向眉心处保持45°角，插入比较通气的鼻孔，速度要慢，如有不适可以保持或退一点，待不适反应不大时再慢慢插入。每个小伙伴的鼻腔情况有差异，只要慢慢找到通道，进入鼻咽部就成功了一半。  （5）反应最大的是咽部的会厌。鼻胃管过了鼻咽就进入了口咽部，这时喉咙会有很大的不适反应，可以喝一口水包在口腔里慢慢地、一点点地吞咽，在吞咽的同时，慢慢地推入鼻胃管，只要鼻胃管过了会厌就基本成功了。  （6）鼻胃管过了会厌后基本会是在食管，为了避免误入气管，可以继续一边喝水一边慢慢地插管，直到插至先前测量的刻度。  （7）为了确保鼻胃管已插到胃里， 可以用准备好的针筒推一点空气至胃里，听听胃里有没有声音，或者用针筒抽一下，看有没有胃液可以抽出来。如果没有，估计还没有过胃上端的贲门，你可以试着再插深一点，直到可以抽出胃液，这样就成功了。  （阿中） | Question 240: Some people are undergoing enteral nutrition therapy with a nasal tube. Is inserting a nasogastric tube dangerous? Can I try it myself?  In fact, nasogastric feeding is not as scary as imagined; it is more of a psychological resistance. Once you mentally accept it, the execution becomes very natural. Nasogastric enteral nutrition has many advantages in terms of implementation and gastrointestinal burden compared to oral enteral nutrition. Typically, inserting a nasogastric tube is done by a doctor. However, in practice, self-insertion is often smoother than when done by a doctor because individuals can feel the angle and position of the gastric tube. They can also time the insertion better and coordinate with swallowing actions. Generally, inserting a gastric tube while awake is unlikely to reach the trachea without any reaction, so self-insertion is entirely feasible and even encouraged. For individuals intolerant to many medications, this may be a method of controlling the disease.  The specific steps for inserting a nasogastric tube are as follows:  Preparation:  Wash hands thoroughly for hygiene.  Prepare a nasogastric tube, syringe, marker pen, adhesive tape (3M is preferable to avoid allergies), scissors, and a glass of water.  Check which nostril is more open to determine which one to use.  Measure the length of the nasogastric tube needed. Measure from the hairline to the xiphoid process or from the tip of the nose to the earlobe and then to the xiphoid process. Make a note of the measurement or mark it on the tube.  Insertion Steps:  Drink a sip of water to lubricate the oral cavity and esophagus.  Let the head of the nasogastric tube hang naturally, finding the naturally curved direction.  Hold the tube 10 centimeters from the head end, keeping the naturally curved direction facing oneself.  Hold the tube at a 45-degree angle towards the forehead, slowly insert it into the more ventilated nostril. Insert slowly and adjust if there is discomfort, continuing when the discomfort is minimal. Once the tube enters the nasopharynx, half of the success is achieved.  The major reaction occurs in the pharynx. Once the tube passes the nasopharynx and enters the oropharynx, there will be significant discomfort in the throat. Drink a sip of water and slowly swallow while pushing the tube, gradually advancing it. The tube is considered successful once it passes the uvula.  After passing the uvula, the tube will be in the esophagus. To avoid entering the trachea, continue to drink water while slowly inserting the tube until it reaches the measured mark.  To ensure the tube is in the stomach, inject a little air into the stomach using the prepared syringe, listen for any sounds in the stomach, or try to aspirate gastric fluid. If unsuccessful, try inserting a little deeper until gastric fluid can be aspirated.  (Ah Zhong) |
| 问题241：我并发了肛周脓肿或肛瘘，我该怎么办？  肛周脓肿和肛瘘都是炎症性肠病常见的并发症。起初，伙伴们大多以为是痔疮，又由于其处在较私密的位置，让我们羞于启齿，甚至以为吃点消炎药或涂些药膏就会痊愈，所以常常会被延误治疗。面对炎症性肠病，我们还是要以积极的态度对待，所以当身体出现任何不适时，都要与你的专科医生联系，让他们给出建议，不要觉得难为情或不好意思。  除了遵医嘱外，这里也把我的经验分享给伙伴们。其实，肛周并发症的根源还是炎症性肠病在发作，所以有效地控制好炎症性肠病对预防和治疗肛周并发症有着非常重要的作用，我们不能捡了芝麻丢了西瓜。在平时要注意保持肛周部位的卫生，勤换内裤（尽量选择宽松透气的那种）。每次便后最好可以温开水坐浴15～20分钟，如果肛周稍有不适则可以在水中加些食盐后坐浴。另外，很多伙伴长期大便不成形，导致肛门括约肌得不到锻炼，所以养成提肛的习惯对我们也非常有利。  （阿中） | Question 241: I have a perianal abscess or fistula. What should I do?  Perianal abscess and fistula are common complications of inflammatory bowel disease. Initially, many individuals often mistake them for hemorrhoids. Due to their location in a more private area, people may be reluctant to discuss them and might believe that taking some anti-inflammatory drugs or applying ointments will lead to recovery. Consequently, treatment is often delayed. In the face of inflammatory bowel disease, it is essential to adopt a proactive attitude. Therefore, when experiencing any discomfort, it is crucial to contact your specialist for advice. Do not feel embarrassed or hesitant.  In addition to following medical advice, I would like to share my experience with you. The root cause of perianal complications lies in the flare-ups of inflammatory bowel disease. Thus, effectively controlling inflammatory bowel disease plays a vital role in preventing and treating perianal complications. We must not overlook the bigger picture. Maintaining hygiene in the perianal area is important, so regularly change underwear (preferably choosing loose and breathable types). After each bowel movement, it is advisable to take a warm sitz bath for 15-20 minutes. If there is any discomfort in the perianal area, adding some salt to the water during the sitz bath may be beneficial. Additionally, many individuals with long-term loose stools fail to exercise the anal sphincter. Therefore, developing a habit of anal contraction is also beneficial.  (Ah Zhong) |
| 问题242：加入炎症性肠病病友的大家庭，彼此相互鼓励、相互帮助对我们来说很重要吗？  人活着最怕的就是孤独，更别说对于一个身患炎症性肠病的人了，那种内心的孤独，那种孤军奋战的疲惫感，那种苦闷的心情，想必伙伴们都曾有过。当我们慢慢地从迷雾中走出来，我想我们应该更有能力和经验去帮助新加入的伙伴们，让他们更快、更好地面对疾病。在帮助他们的同时，我们自己也可以从中获益，你会见到更多、更复杂的情况，也会了解到更多、更好的办法。因为疾病在发展，治疗疾病的方式方法也在不停地更新发展。我们只要保持疾病的缓解，就一定会等到攻克炎症性肠病的那一天。  （阿中） | Question 242: Is joining the big family of inflammatory bowel disease patients, encouraging and helping each other, important for us?  The fear of loneliness is one of the greatest challenges in life, especially for someone with inflammatory bowel disease. The internal loneliness, the exhaustion of fighting alone, and the oppressive feelings are experiences many of us have faced. As we gradually emerge from the mist, I believe we should have the ability and experience to help newcomers in the community, enabling them to face the disease more quickly and effectively. While assisting them, we ourselves can also benefit. You will encounter more diverse and complex situations and gain insights into more effective approaches. As the disease evolves, so do the methods and techniques for treating it. As long as we maintain disease remission, the day when we conquer inflammatory bowel disease will surely come.  (Ah Zhong) |
