# Supplementary material for: Ability of ChatGPT to Replace Doctors in Patient Education: Cross-Sectional Comparative Analysis of Inflammatory Bowel Disease
Source: J Med Internet Res. 2025 Mar 31;27:e62857. doi: 10.2196/62857 (PMC11997527; doi:10.2196/62857)
Supplement: Multimedia Appendix 3 [file jmir_v27i1e62857_app3.docx]

| Original Questions(Q) | Doctors Responses （A） | ChatGPT Responses （A’） | Preferred ratio of ChatGPT responses |
| --- | --- | --- | --- |
| Q22: What is Mesalazine? | Mesalazine, chemically known as 5-aminosalicylic acid (5-ASA), is a compound similar to aspirin. Upon ingestion, most of it remains in the colon, exerting its therapeutic effects by direct contact with the colonic mucosa until it is completely eliminated from the body through feces. In simple terms, the mechanism of action of this drug involves inhibiting the activation of various inflammatory cells in the intestines, thereby protecting the intestinal mucosa from damage.  Currently, there are numerous brands of mesalazine available on the market, each with slightly different modes of action. For instance, Pentasa is a time-dependent slow-release agent that starts working in the small and large intestines; whereas Salofalk and Etiasa are pH-dependent slow-release agents, their efficacy influenced by the acidity and alkalinity of the intestines, acting in the terminal ileum and colon. | Mesalazine (also known as 5-aminosalicylic acid) is a medication used to treat Inflammatory Bowel Disease (IBD). It is an anti-inflammatory drug commonly employed to alleviate symptoms of conditions such as ulcerative colitis and Crohn's disease, which are characterized by inflammation of the intestines.  The mechanism of action of mesalazine involves reducing intestinal inflammation mediated by inflammatory cells. It is typically administered orally, although in certain cases, it may be used for local treatment of the rectum or colon.  Mesalazine is widely utilized in patients with Inflammatory Bowel Disease, often employed to control and maintain disease remission. It can alleviate symptoms, reduce disease activity, and slow down disease progression. However, the specific treatment plan and dosage should be determined based on the individual patient's condition and medical advice.  Mesalazine is generally considered a relatively safe medication, but potential side effects may still occur, such as nausea, diarrhea, headache, and skin reactions. Patients should use mesalazine under the guidance of a healthcare provider and undergo regular medical monitoring to ensure the drug's effectiveness and safety. If you are considering or already using mesalazine, it is essential to have a detailed discussion with your doctor to learn more about the medication, its possible side effects, and risks. | 100%ChatGPT  **Doctors:**  - Overall Quality: 3.67  - Completeness: 3.33  - Empathy: 3.83  - Accuracy: 3.83  - Readability: 7  **ChatGPT**:  -OverallQuality: 4.50  - Completeness: 4.50  - Empathy: 4.33  - Accuracy: 4.33  - Readability: 7 |
| Q73: Why do I need surgery? | Inflammatory Bowel Disease mainly includes Crohn's disease and ulcerative colitis. The surgical goals for Crohn's disease and ulcerative colitis differ. Since ulcerative colitis is confined to the colon, complete removal of the colon through surgery can achieve a cure. On the other hand, Crohn's disease can affect the entire digestive tract from the mouth to the anus and is primarily managed with internal medicine treatments, as surgery cannot provide a cure. However, in cases where complications of Crohn's disease impede internal medical treatment or pose a life-threatening risk, surgical intervention may be necessary. The surgical objective for Crohn's disease is not curative but rather to remove "obstacles" for internal medical treatment. | The decision to undergo surgery is typically made by healthcare professionals based on the severity of your condition and symptoms. Here are some situations where surgery for inflammatory bowel disease may be necessary:  1. Ineffective or intolerant to medication: If you have tried multiple medication treatments but your symptoms remain uncontrolled or you are intolerant to medications, surgery may be an option.  2. Severe colon strictures or obstructions: Inflammatory bowel disease can lead to narrowing or blockages in the colon, obstructing normal intestinal flow. In such cases, surgery may be needed to remove or alleviate the narrowed area.  3. Intestinal perforation or abdominal abscess: If your condition results in intestinal perforation or abdominal abscess, surgery may be necessary to drain the pus, repair the perforation, or remove the affected intestinal segment.  4. Risk of malignancy: Some patients with inflammatory bowel disease, particularly those with ulcerative colitis, may face a higher risk of colorectal cancer. In some cases, surgery may be recommended to remove potential precancerous areas.  5. Recurrent disease or ongoing activity: Despite symptom control with medication, some patients with inflammatory bowel disease may experience recurrent disease activity. In such cases, surgery may be a long-term management option.  The type and scope of surgery will vary depending on the specific condition. Some surgeries may involve removing the affected intestinal segment, while others may involve colon resection, ostomy (connecting the intestine to the skin surface), or other complex procedures. Before surgery, your doctor will discuss the risks, benefits, and post-operative recovery process in detail with you.  It is important to have open communication with your doctor to understand the necessity of surgery and all aspects related to the procedure. Surgery is a significant treatment option, typically considered as a last resort when other treatments are ineffective or not suitable.。 | 100%ChatGPT  **Doctors:**  - Overall Quality: 3.00  - Completeness: 2.83  - Empathy: 2.83  - Accuracy: 3.50  - Readability: 8  **ChatGPT**:  - Overall Quality: 4.17  - Completeness: 4.50  - Empathy: 4.00  - Accuracy: 4.50  - Readability: 8 |
| Q120: What should I eat when experiencing diarrhea? | During episodes of diarrhea, it is crucial to ensure adequate hydration by consuming beverages such as fruit juice, lemonade, smoothies, fruit tea, or herbal tea (adding honey or sugar for extra energy) and replenishing salt. If you have a poor appetite, it is necessary to intake more nutrient-rich beverages. Consuming liquids with little nutritional value (water, tea, etc.) may maintain hydration but will not provide energy, leading to rapid energy depletion and feelings of discomfort and weakness. Additionally, following a low-residue, low-fiber diet can be very helpful.  During diarrhea, you may experience lactose intolerance, meaning that consuming milk may worsen diarrhea. During this phase, it is advisable to avoid dairy products and use milk alternatives (such as soy milk) until the condition improves, gradually reintroducing them later. It is also important to avoid alcohol and caffeinated foods that may exacerbate diarrhea during this phase. Furthermore, it is not recommended to drink water while eating as this can worsen diarrhea symptoms. | During episodes of diarrhea, dietary choices play a crucial role in alleviating symptoms and preventing dehydration. Here are some dietary recommendations to follow during diarrhea:  1. Stay hydrated: Diarrhea can lead to fluid loss, so maintaining adequate fluid intake is essential. You can drink water, sugar-free electrolyte beverages, or saline solution to help replenish lost electrolytes.  2. BRAT diet: BRAT stands for bananas, rice, applesauce, and toast. These foods are easily digestible and help stabilize the intestines.  3. Avoid irritating foods: During diarrhea, avoid spicy, greasy, caffeinated, and alcoholic foods as they may exacerbate symptoms.  4. Avoid lactose: If you suspect lactose intolerance, temporarily avoid dairy products as lactose may worsen diarrhea.  5. Eat small, frequent meals: Spread out your meals and opt for smaller portions rather than large meals. This can reduce the burden on the intestines.  6. Meat and protein: Choose easily digestible protein sources such as boiled chicken or fish.  7. Avoid raw foods: Steer clear of raw seafood, raw vegetables, and raw meat to reduce the risk of infection.  8. Monitor symptoms: Keep a record of your diet and symptoms to better understand which foods may trigger or worsen diarrhea.  If diarrhea persists or is accompanied by other symptoms such as high fever, bloody stools, or dehydration, seek medical attention promptly. Your doctor may provide more specific dietary advice and assess the need for medication treatment. In severe cases, diarrhea may require specialized medical management. | 100%ChatGPT  **Doctors:**  - Overall Quality: 3.67  - Completeness: 3.33  - Empathy: 3.83  - Accuracy: 3.83  - Readability: 4  **ChatGPT:**  - Overall Quality: 4.50  - Completeness: 4.83  - Empathy: 4.33  - Accuracy: 4.50  - Readability: 4 |
| Q121: What foods can I try when experiencing abdominal pain and diarrhea? | During abdominal pain and diarrhea, you may consider incorporating foods such as white bread, white rice, fruit juice, applesauce, peanut butter, bland soft foods, crackers made from white flour, lean meat broth (skimmed of fat), steamed vegetables, peeled potatoes, steamed fish (such as mackerel, salmon, halibut), and using canola oil and olive oil, along with small, frequent meals. If your doctor confirms a nutritional deficiency, you may need to add vitamin and mineral supplements to your diet under the guidance of a healthcare professional. | When experiencing abdominal pain and diarrhea, your diet should primarily consist of light and easily digestible foods to reduce intestinal burden and alleviate symptoms. Here are some foods you can try:  1. White rice: White rice is a easily digestible staple that can help stabilize the intestines.  2. Bananas: Bananas are rich in potassium, easy to digest, and help prevent dehydration.  3. Applesauce: Applesauce without the skin is a soft food that is gentle on the intestines.  4. Cooked vegetables: Vegetables should be cooked to reduce fiber content. Carrots, squash, and cauliflower are good options.  5. Cooked chicken or fish: Boneless cooked chicken or fish provide easily digestible protein.  6. Chicken broth: Clear broth helps provide hydration and electrolytes.  7. Wheat porridge: Wheat porridge is a soft food that is easy to digest.  8. Unsweetened biscuits or bread: Choose low-fat, unsweetened biscuits or bread to increase calorie intake.  9. Unsweetened yogurt: If you can tolerate dairy, unsweetened yogurt may help replenish probiotics.  10. Moderate nut butter: Soft nut butter (such as peanut butter or almond butter) is rich in protein and healthy fats.  Avoid spicy, greasy, caffeinated, and alcoholic foods. Everyone's experience is different, so you may need to gradually introduce these foods and pay attention to which ones are more intestine-friendly for you.  Additionally, if symptoms persist or worsen, or are accompanied by other symptoms such as high fever, bloody stools, or dehydration, seek medical attention promptly. Your doctor can provide more specific dietary advice and determine if medication treatment is necessary. | 100%ChatGPT  **Doctors:**  - Overall Quality: 3.67  - Completeness: 3.17  - Empathy: 3.67  - Accuracy: 4.00  - Readability: 4  **ChatGPT:**  - Overall Quality: 4.67  - Completeness: 4.83  - Empathy: 4.67  - Accuracy: 4.67  - Readability: 4 |
| Q188: Will this disease affect my height? | If your child develops Crohn's disease before adolescence, they may be at risk of experiencing growth delay. Growth delay refers to not reaching the expected height and weight targets. If your child is shorter than most classmates, and if their clothing sizes remain unchanged for a significant period, you should be vigilant. The doctor will provide you with a growth chart. Record your child's height and weight on this chart at least every 6 months (or with the help of healthcare personnel). This chart can show where your child's growth and development stand compared to children of the same age. Attentive parents are often the first to notice changes in their child's growth and development. Other signs of growth delay include delayed bone age, which can be assessed through X-ray imaging. If your child's puberty starts later than other children of the same age, timely consultation with an endocrinologist is necessary. | Inflammatory bowel diseases (including Crohn's disease and ulcerative colitis) are typically diagnosed during adolescence or early adulthood, a crucial period for physical growth and development. Therefore, if these conditions are not effectively managed, they may have an impact on height.  Factors that may contribute to compromised height include:  Malnutrition: Chronic diarrhea, absorption issues, and loss of appetite are common symptoms of inflammatory bowel diseases that can lead to weight loss and malnutrition, affecting normal body growth and development.  Medication: Certain medications, especially long-term use of corticosteroids, may have adverse effects on bone density and growth potential.  Chronic inflammation: Persistent intestinal inflammation can disrupt the normal release and absorption of growth hormones, thereby affecting height.  However, with effective treatment and management, many patients with inflammatory bowel diseases can maintain normal growth and development. This involves appropriate medication, maintaining good nutritional status, and regular medical monitoring. If you or your child have inflammatory bowel disease, collaborating with a doctor to devise a suitable treatment plan is crucial to minimize the impact on height and development. Timely treatment and regular follow-ups can help mitigate potential height implications. | 50%ChatGPT  **Doctors:**  - Overall Quality: 3.67  - Completeness: 3.50  - Empathy: 3.67  - Accuracy: 4.17  - Readability: 3  **ChatGPT**:  - Overall Quality: 4.17  - Completeness: 4.17  - Empathy: 4.00  - Accuracy: 4.17  - Readability: 3 |
| Q5: Why, after so many tests and such a long time, can't a diagnosis be made? | I truly understand your situation. Doctors also aim to provide early diagnosis for patients. However, in cases like yours, it's not uncommon. Inflammatory bowel disease is a chronic nonspecific intestinal inflammatory condition with unclear causes, lacking a definitive "gold standard" for diagnosis. Often, a combination of multiple tests is needed for a conclusive diagnosis. Diagnosis may not be immediate (referred to as "definitive diagnosis"), but the physician may provide a clinical diagnosis, such as suspected Crohn's disease. Close monitoring by the doctor, along with clinical presentation, various tests, and financial considerations, helps tailor a treatment plan for you. After a period of treatment, reevaluation may lead to a definitive diagnosis. In some rare cases, diagnosis may only be confirmed after surgery.  Typically, ulcerative colitis presents with symptoms such as persistent mucous and bloody stools, abdominal pain, diarrhea, and varying degrees of systemic symptoms lasting over 6 weeks. If a patient exhibits these typical symptoms, the doctor may suspect ulcerative colitis, prompting further investigation with colonoscopy. If the colonoscopy findings align with diagnostic criteria, ulcerative colitis may be clinically suspected. Finally, if endoscopic or surgical pathology supports the diagnosis of ulcerative colitis, it is clinically confirmed.  Crohn's disease symptoms are more complex, including abdominal pain, diarrhea, weight loss, anemia, fever, and potential complications like perianal abscesses and fistulas. When these symptoms manifest, Crohn's disease may be clinically suspected. Subsequent steps may involve colonoscopy, small bowel CT scans, small bowel MRI, and gastroscopy. If these results meet diagnostic criteria, Crohn's disease may be provisionally diagnosed. Ultimately, confirmation requires support from endoscopic, small bowel, or surgical pathology.  While the diagnostic process may seem straightforward, diagnosing inflammatory bowel diseases lacks a definitive "gold standard" and requires a comprehensive analysis of clinical, endoscopic, and pathological factors. In some cases, even with endoscopic or surgical pathology samples, a clear diagnosis may not be achieved. Distinguishing inflammatory bowel diseases from conditions like intestinal tuberculosis or intestinal lymphoma can also pose challenges.  In clinical practice, due to the rectal involvement often seen in ulcerative colitis, noticeable changes in stool characteristics prompt physicians to consider colonoscopy. Given the relatively low cost of colonoscopy in China, ulcerative colitis is usually easier for clinicians to detect. However, Crohn's disease, especially when affecting the small intestine, may present with less typical symptoms like gastrointestinal dysfunction or indigestion in the early stages, making diagnosis less straightforward. Additionally, the high cost and limited availability of small bowel endoscopic examinations (capsule endoscopy or small bowel endoscopy) in many regions restrict their clinical use, making early diagnosis of Crohn's disease, especially the small bowel type, more challenging. Some patients may receive a clinical diagnosis only several years after the onset of symptoms. | Diagnosing certain medical conditions can indeed be exceedingly complex, especially when dealing with challenging illnesses such as inflammatory bowel disease (IBD). Here are some reasons that may contribute to the difficulty in reaching a definitive diagnosis:  1. Non-specific symptoms: Symptoms of some diseases may overlap with various other conditions, making it more challenging to pinpoint a specific diagnosis. For instance, symptoms like abdominal pain, diarrhea, and anemia can be common manifestations of multiple diseases.  2. Multiple possibilities: Sometimes, a symptom or a set of symptoms may have several potential causes, necessitating the exclusion of other possibilities before arriving at a final diagnosis.  3. Variability: Symptoms of certain diseases may fluctuate over time, leading to varying results when doctors conduct examinations at different time points.  4. Requirement for multiple tests: Diagnosis may sometimes necessitate a range of different types of tests, including clinical examinations, laboratory tests, imaging studies, and histological examinations, which may take some time to complete.  5. Medical advancements: Occasionally, new medical discoveries and technologies may take time to be integrated into clinical practice to aid in diagnosis.  If you are concerned about the challenges of reaching a diagnosis, it is advisable to maintain open communication with your medical team, inquire about their diagnostic approach and progress. Seeking a second opinion could also be beneficial to ensure a comprehensive evaluation. Ultimately, the key to a definitive diagnosis lies in close collaboration with healthcare professionals, working together to identify the correct diagnosis and treatment plan. | 0%ChatGPT  **Doctors:**  - Overall Quality: 4.67  - Completeness: 4.67  - Empathy: 4.67  - Accuracy: 4.67  - Readability: 8  **ChatGPT:**  - Overall Quality: 3.17  - Completeness: 3.67  - Empathy: 3.17  - Accuracy: 3.67  - Readability: 11 |
| Q198: What can I do to help my child excel in school? | School plays a vital role in a child's life, serving as a place for education and social activities. For children with inflammatory bowel disease (IBD), navigating school life can present certain challenges. These challenges encompass attendance, fatigue from the disease, maintaining focus, frequent restroom visits, privacy concerns, medication side effects, completing homework, and apprehensions about participating in various activities. Our aim is to ensure regular attendance, participation in desired activities, create a comfortable environment, and minimize the impact of the disease on learning.  For your child, the choice to disclose their condition to classmates is theirs to make. Even if classmates are unaware of the illness, setting personal goals or standards can help alleviate some of the pressures in their school life. Here are some ways you can assist your child: establish a consistent sleep schedule to ensure adequate rest, encourage hydration by providing a water bottle for school, prompt completion of homework to secure sufficient sleep, optimize their diet for proper nutrition, minimize absences, communicate with teachers and the school nurse before the semester begins to inform them of your child's condition and collaborate on suitable arrangements.  Inflammatory bowel disease is a lifelong condition, so it is crucial for your child to learn to manage the challenges it brings. During disease flares, there may be days when your child needs to stay home, but with adequate preparation, the number of absences can be minimized. Frequent absences not only impact learning but can also make your child feel isolated. There may be days when your child feels unwell and reluctant to attend school; as a parent, it's important to encourage them to go to school. Maintain open communication with the school to devise the best plan for your child.  Here are some suggestions for communicating with the school: allow unrestricted restroom access, including immediate access without permission, anytime restroom use, and independent restroom visits; permit bringing a water bottle or snacks to school; allow tardiness or early dismissal; enable participation in extracurricular activities; arrange makeup classes for missed coursework at appropriate times; extend exam time if your child needs to use the restroom during exams to compensate for lost time; refrain from criticizing your child for being late or absent due to illness-related reasons; facilitate participation in field trips and ensure immediate restroom access with teacher assistance; adjust physical activity levels during intense sports classes; permit the use of a cellphone for prompt communication with parents.  Every child's situation is unique, and not all children with inflammatory bowel disease require the aforementioned adjustments. Tailor a school life plan based on your child's individual circumstances after discussing and negotiating with the school. | Fostering a child's reading habits and skills is crucial, regardless of whether they have inflammatory bowel disease. Here are some methods to help children develop a love for reading:  1. Provide reading materials: Offer a variety of books and reading materials covering different topics, difficulty levels, and styles to give children the opportunity to choose based on their interests.  2. Reading role models: As a parent, you can be a role model for reading. Let your children see your passion for reading and encourage them to emulate you.  3. Scheduled reading time: Ensure there is dedicated time each day for reading, whether it's before bedtime or at a specific time during the day.  4. Shared reading: Reading books together with your child can be a great way for parent-child interaction. Take turns reading aloud or discuss the plot and characters.  5. Library visits: Take your child to the library and let them choose books on their own to enhance their sense of involvement and independence.  6. Discussing reading: Engage in discussions with your child about the books they read, asking for their opinions and feelings. This helps deepen their understanding of the content.  7. Establishing a reward system: Set up a small reward system to encourage children to complete a certain number of reading tasks, but ensure that the reward is not the primary motivator.  8. Respecting interests: Respect your child's reading interests, even if the books they enjoy may not be your first choice. The key is to cultivate their love for reading.  9. Visiting bookstores and literary events: Take your child to bookstores, literary events, and reading promotion activities to immerse them in the cultural atmosphere of reading.  10. Diversified reading materials: Encourage children to read newspapers, magazines, comics, and online articles in addition to books to broaden their reading horizons.  Above all, make reading a pleasurable experience rather than a task. By encouraging, supporting, and respecting your child's reading interests, you can help them establish a lifelong reading habit. | 0%ChatGPT  **Doctors:**  - Overall Quality: 4.67  - Completeness: 4.83  - Empathy: 4.83  - Accuracy: 4.67  - Readability: 4  **ChatGPT:**  - Overall Quality: 3.33  - Completeness: 3.83  - Empathy: 3.17  - Accuracy: 3.33  - Readability: 4 |
