# Supplementary material for: Ability of ChatGPT to Replace Doctors in Patient Education: Cross-Sectional Comparative Analysis of Inflammatory Bowel Disease
Source: J Med Internet Res. 2025 Mar 31;27:e62857. doi: 10.2196/62857 (PMC11997527; doi:10.2196/62857)
Supplement: Multimedia Appendix 4 [file jmir_v27i1e62857_app4.docx]

“"AI hallucinate"

In cases where the text is off-topic, we will ask for clarification three times, providing as much detailed background information as possible; To avoid mistakenly rejecting ChatGPT's response due to insufficient prompts or context setting.

1.Example questions and answers on two commonly used medications for Inflammatory bowel disease

| Q27.艾迪莎和颇得斯安在哪里释放？原理是什么？  "艾迪莎"（Aedes aegypti）和 "颇得斯安"（Aedes albopictus）是两种蚊子的学名，它们是传播病毒的蚊子，其中包括黄热病、登革热和寨卡病毒等。这两种蚊子通常在温暖地区和热带地区被发现，尤其是在亚洲、非洲、拉丁美洲和一些美国地区。  这些蚊子传播病毒的原理是它们叮咬感染了病毒的宿主（通常是人类），然后将病毒带入它们的体内。当这些蚊子再次叮咬另一个人时，它们会将病毒通过唾液传播给新的宿主。这种传播病毒的方式被称为生物传播。  以下是一些相关疾病的简要说明：  * 黄热病（Yellow Fever）： 由黄热病病毒引起，通过感染的"Aedes aegypti"蚊子传播给人类。黄热病可以导致高热、头痛、肌肉疼痛和黄疸等症状，严重情况下可能致命。  * 登革热（Dengue Fever）： 由登革病毒引起，通过感染的"Aedes aegypti"和"Aedes albopictus"蚊子传播给人类。登革热的症状包括高热、头痛、肌肉和关节疼痛、皮疹等，可能引发严重并发症。  * 寨卡病毒（Zika Virus）： 由寨卡病毒引起，通过感染的"Aedes aegypti"和"Aedes albopictus"蚊子传播给人类。寨卡病毒感染可能引发发热、皮疹、关节痛和结膜炎等症状，对孕妇和胎儿可能有严重影响。  为了预防这些疾病的传播，控制"Aedes aegypti"和"Aedes albopictus"蚊子的繁殖和传播至关重要。这包括采取措施减少蚊子的繁殖地点，如清除积水容器、使用蚊帐、使用驱蚊剂等方法。此外，疫苗也可以用于一些疾病的预防，如黄热病疫苗和登革热疫苗。 | Q27.Where were ETIASA and Pentasa released? What is the mechanism?  "ETIASA^（^Aedes aegypti）" and "Pentasa^（^Aedes albopictus）" are two species of mosquitoes, known for transmitting viruses such as yellow fever, dengue fever, and Zika virus. These mosquitoes are typically found in warm and tropical regions, particularly in Asia, Africa, Latin America, and some parts of the United States.  The principle behind how these mosquitoes transmit viruses is that they bite an infected host (usually a human) and then carry the virus within their bodies. When they bite another person, they transmit the virus to the new host through their saliva. This mode of virus transmission is known as biological transmission.  Here is a brief overview of some related diseases:  Yellow Fever: Caused by the yellow fever virus, transmitted to humans by infected "Aedes aegypti" mosquitoes. Yellow fever can lead to symptoms such as high fever, headache, muscle pain, and jaundice, and in severe cases, it can be fatal.  Dengue Fever: Caused by the dengue virus, transmitted to humans by infected "Aedes aegypti" and "Aedes albopictus" mosquitoes. Symptoms of dengue fever include high fever, headache, muscle and joint pain, rash, and potentially severe complications.  Zika Virus: Caused by the Zika virus, transmitted to humans by infected "Aedes aegypti" and "Aedes albopictus" mosquitoes. Zika virus infection can lead to symptoms such as fever, rash, joint pain, and conjunctivitis, with potentially serious implications for pregnant women and fetuses.  To prevent the spread of these diseases, it is crucial to control the breeding and spread of "Aedes aegypti" and "Aedes albopictus" mosquitoes. This includes measures to reduce mosquito breeding sites, such as eliminating standing water containers, using mosquito nets, and applying insect repellents. Additionally, vaccines can be used for the prevention of some diseases, such as yellow fever vaccine and dengue fever vaccine. | *Original Question*  First response |
| --- | --- | --- |
| 我问的是美沙拉嗪的两种药剂制型，艾迪莎和颇得斯安。  非常抱歉，我之前的回答存在误解。"艾迪莎"和"颇得斯安"这两个名词与美沙拉嗪（Mesalazine）或与药物制剂无关，似乎存在一些混淆。  美沙拉嗪通常以不同的商品名在不同国家销售，因此在不同地方可能有不同的药品品牌名称。"艾迪莎"和"颇得斯安"这两个名词似乎不是与美沙拉嗪相关的常见品牌或制剂名称。  如果您对美沙拉嗪的不同制剂或品牌有疑问，建议您咨询您的医生或药剂师，以获取关于可用的药物制剂的具体信息以及哪种适合您的情况。他们可以提供有关可用药物的详细信息，以便您做出明智的药物选择。 | I'm asking about the two pharmaceutical formulations of mesalazine, ETIASA and Pentasa  I deeply apologize for the misunderstanding in my previous response. The terms "ETIASA " and "Pentasa" do not seem to be related to Mesalazine or any medication formulations, leading to some confusion. Mesalazine is typically marketed under different brand names in different countries, hence there may be variations in drug brand names in different regions. The terms "Aidisha" and "Podessaan" do not appear to be common brand or formulation names associated with Mesalazine. If you have any inquiries regarding different formulations or brands of Mesalazine, it is advisable to consult with your physician or pharmacist for specific information on available medication formulations and which would be suitable for your condition. They can provide detailed information on the available medications to assist you in making an informed choice. | *Continue the questioning, supplement the hints.*  Second response |
| 你真得不知道，艾迪莎和颇得斯安在哪里释放吗？以及它们原理是什么？  非常抱歉，我之前的回答还是存在误解。"艾迪莎"和"颇得斯安"这两个名词在我之前的回答中没有相关信息，我也无法提供关于它们的详细信息，因为它们可能不是通用的药物或制剂名称，或者可能是某些地区特有的药物。  如果您有关于特定药物或制剂的疑问，我建议您咨询您的医生、药剂师或健康专家。他们可以为您提供有关这些药物的详细信息，包括它们的释放地点和作用原理。同时，确保提供药物的正确名称或品牌名称，以便获得准确的信息。再次为之前的回答不准确而道歉，希望您能找到您需要的信息。 | Do you really not know where ETIASA and Pentasa are released? And what are their principles?  I apologize for the misunderstanding in my previous response. The nouns "ETIASA" and "Pentasa " did not have any relevant information in my previous answer, and I am unable to provide detailed information about them as they may not be common drug or formulation names, or they could be specific to certain regions.  If you have questions about specific drugs or formulations, I recommend consulting your doctor, pharmacist, or healthcare professional. They can provide you with detailed information about these drugs, including their release locations and mechanisms of action. Additionally, make sure to provide the correct name or brand name of the drug to obtain accurate information. I apologize again for the inaccuracies in my previous response and hope you find the information you need. | *Continue the questioning, supplement the hints once more..*  Third response |

2.Example questions and answers on specific context, the use of highly colloquial terms as cue words.

"激素”（Hormones） is the abbreviation of “糖皮质激素”（Glucocorticoids） in Chinese, and they are often used interchangeably in clinical medical settings.

| Q28.激素是什么？  激素（Hormones）是人体内分泌系统中的化学信使，它们由内分泌腺体（如甲状腺、肾上腺、胰岛、性腺等）分泌，并通过血液传播到身体的不同部位，以调节和协调各种生理过程和功能。激素在人体中扮演重要的角色，影响到几乎所有生理和代谢活动。  以下是一些常见的激素以及它们的作用：  * 胰岛素（Insulin）： 由胰腺分泌，帮助调节血糖水平，促使细胞摄取葡萄糖并将其转化为能量。  * 甲状腺激素（Thyroid Hormones）： 由甲状腺分泌，影响代谢率、体温和生长发育。  * 皮质醇（Cortisol）： 由肾上腺分泌，参与应激应答、免疫调节和蛋白质、脂肪、碳水化合物代谢。  * 雌激素和孕激素（Estrogens and Progestogens）： 由卵巢分泌，控制女性的生殖周期、性征发育和妊娠。  * 睾丸激素（Testosterone）： 由睾丸分泌，控制男性的性征发育、生殖功能和骨密度。  * 肾上腺素和去甲肾上腺素（Epinephrine and Norepinephrine）： 由肾上腺分泌，参与应激应答，如“战斗或逃跑”反应，提高心率和血压。  * 生长激素（Growth Hormone）： 由垂体分泌，促进生长和修复组织。  * 抗利尿激素（Antidiuretic Hormone，也称为ADH或vasopressin）： 由垂体分泌，控制体液平衡和血容量。  这些激素通过与身体的受体相互作用，以维持身体内部环境的稳定性（称为内稳态）并调整生理功能。激素的不平衡或异常分泌可能导致各种健康问题和疾病，因此激素的平衡对于维持健康非常重要。激素疾病的治疗通常涉及药物疗法或其他治疗方法，以调整激素水平并恢复正常的生理功能。 | Q28. What are “激素”（hormones）?  Hormones are chemical messengers in the endocrine system of the human body. They are secreted by endocrine glands such as the thyroid, adrenal glands, pancreas, and sex glands, and circulate through the blood to various parts of the body to regulate and coordinate various physiological processes and functions. Hormones play a crucial role in the human body, influencing nearly all physiological and metabolic activities.  Here are some common hormones and their functions:  - Insulin: Secreted by the pancreas, helps regulate blood sugar levels by promoting the uptake of glucose by cells and converting it into energy.  - Thyroid Hormones: Secreted by the thyroid gland, influence metabolism rate, body temperature, and growth and development.  - Cortisol: Secreted by the adrenal glands, involved in stress response, immune regulation, and protein, fat, and carbohydrate metabolism.  - Estrogens and Progestogens: Secreted by the ovaries, control the female reproductive cycle, development of secondary sexual characteristics, and pregnancy.  - Testosterone: Secreted by the testes, controls male secondary sexual characteristics, reproductive function, and bone density.  - Epinephrine and Norepinephrine: Secreted by the adrenal glands, involved in stress response, such as the "fight or flight" response, increasing heart rate and blood pressure.  - Growth Hormone: Secreted by the pituitary gland, promotes growth and tissue repair.  - Antidiuretic Hormone (ADH or vasopressin): Secreted by the pituitary gland, controls fluid balance and blood volume.  These hormones interact with receptors in the body to maintain internal environment stability (known as homeostasis) and regulate physiological functions. Imbalance or abnormal secretion of hormones can lead to various health problems and diseases, making hormone balance crucial for maintaining health. Treatment of hormone disorders typically involves medication therapy or other treatment methods to adjust hormone levels and restore normal physiological functions. | *Original Question*  First response |
| --- | --- | --- |
| 糖皮质激素是什么？  糖皮质激素（Glucocorticoids）是一类由肾上腺分泌的激素，它们在人体内发挥着多种重要的生理作用。这些激素包括皮质醇（Cortisol）等，它们的名字中包含"糖皮质"（Glucocorticoid）表明它们对糖代谢（即葡萄糖代谢）有重要影响。  糖皮质激素的主要作用包括：  * 调节糖代谢： 糖皮质激素可以促使肝脏释放葡萄糖到血液中，以增加血糖水平。这对于在应激情况下提供额外的能量非常重要。  * 抗炎作用： 糖皮质激素具有强烈的抗炎和免疫抑制作用。它们可以减轻炎症反应和过敏反应，因此常用于治疗炎症性疾病，如风湿性关节炎、哮喘、过敏性皮肤炎等。  * 免疫抑制： 糖皮质激素可以抑制免疫系统的活动，降低免疫反应，因此它们常用于器官移植术后预防器官排斥反应，以及治疗自身免疫性疾病。  * 抗过敏作用： 糖皮质激素可以减轻过敏反应，如过敏性鼻炎和皮肤过敏。  尽管糖皮质激素在治疗许多疾病中非常有效，但它们也具有一些潜在的副作用和风险，特别是在长期或高剂量使用时。一些常见的副作用包括骨密度下降、体重增加、高血压、糖尿病、皮肤问题和免疫系统抑制。  因此，在使用糖皮质激素时，必须严格遵循医生的建议和处方，以确保安全和有效的治疗。此外，长期使用后通常需要逐渐减少剂量，而不是突然停止，以避免戒断症状和复发。如果您有关于糖皮质激素的使用或其副作用的疑问，建议您咨询医生，他们将能够为您提供更详细的信息和指导。 | What are “糖皮质激素”（Glucocorticoids）?  Glucocorticoids are a class of hormones secreted by the adrenal glands that play various important physiological roles in the human body. These hormones, including cortisol, have names containing "glucocorticoid," indicating their significant impact on glucose metabolism.  The primary functions of glucocorticoids include:  - Regulating glucose metabolism: Glucocorticoids can stimulate the liver to release glucose into the bloodstream to increase blood sugar levels, which is crucial for providing additional energy in stressful situations.  - Anti-inflammatory effects: Glucocorticoids have potent anti-inflammatory and immunosuppressive effects. They can alleviate inflammatory and allergic reactions, making them commonly used in treating inflammatory conditions such as rheumatoid arthritis, asthma, and allergic dermatitis.  - Immunosuppression: Glucocorticoids can suppress the activity of the immune system, reducing immune responses. Therefore, they are often used to prevent organ rejection after organ transplantation and treat autoimmune diseases.  - Anti-allergic effects: Glucocorticoids can alleviate allergic reactions, such as allergic rhinitis and skin allergies.  Although glucocorticoids are highly effective in treating many diseases, they also carry some potential side effects and risks, especially with long-term or high-dose use. Common side effects include decreased bone density, weight gain, high blood pressure, diabetes, skin issues, and immune system suppression.  Therefore, when using glucocorticoids, it is essential to strictly follow the doctor's advice and prescription to ensure safe and effective treatment. Additionally, gradual dose reduction is typically required after long-term use instead of abrupt cessation to prevent withdrawal symptoms and relapse. If you have any questions about the use of glucocorticoids or their side effects, it is recommended to consult a doctor, as they will be able to provide you with more detailed information and guidance. | *Continue the questioning, supplement the hints.*  Second response |
