# Supplementary material for: Ability of ChatGPT to Replace Doctors in Patient Education: Cross-Sectional Comparative Analysis of Inflammatory Bowel Disease
Source: J Med Internet Res. 2025 Mar 31;27:e62857. doi: 10.2196/62857 (PMC11997527; doi:10.2196/62857)
Supplement: Multimedia Appendix 5 [file jmir_v27i1e62857_app5.docx]

Q1.Why do I have this strange disease called Crohn's disease?

It is evident that while the specific wording varies in each version, the structure and informational content do not differ significantly.

| First edition | Second edition | Third edition |
| --- | --- | --- |
| Crohn's disease is a chronic inflammatory bowel disease, the exact cause of which remains unclear, but research suggests it may be the result of multiple interacting factors. While we cannot provide personalized medical advice, here are some potential factors and risk factors that may contribute to Crohn's disease: (conceptual clarification, open dialogue)  1.Genetic factors: Individuals with a family history may be more susceptible to Crohn's disease. Genetic factors may play a role in the onset of this disease.  2.Immune system issues: Crohn's disease is believed to be a result of an abnormal immune system response, where the immune system may mistakenly attack normal cells and bacteria in the intestines, leading to inflammation.  Environmental factors: Some environmental factors, such as smoking, diet, infections, and exposure to certain chemicals, may be associated with the development of Crohn's disease.  3.Intestinal microbiota: The microbial community in the intestines may play a role in the development of Crohn's disease. Imbalances in the microbiota may trigger inflammatory responses.  4.Other factors: Psychological stress, substance abuse, autoimmune diseases, and other factors may also increase the risk of developing Crohn's disease.  (elaboration of content)  It is important to remember that the exact causes of Crohn's disease vary from person to person, and each individual may be influenced by different factors. If you suspect you have Crohn's disease or experience related symptoms, it is advisable to consult a doctor for professional diagnosis and treatment advice. Doctors will determine whether you have Crohn's disease based on your medical history, physical examination, and possible diagnostic tests, and provide appropriate treatment options. (open dialogue, professional guidance) | Crohn's disease is a type of chronic inflammatory bowel disease (IBD) with an unclear exact cause, but likely involving interactions among various factors such as genetics, the immune system, environment, and lifestyle. While research is ongoing, the following factors may be associated with the onset of Crohn's disease: (conceptual clarification)  1. Genetic factors: Individuals with a family history have a higher risk of developing Crohn's disease. Studies have identified certain genetic variations that may increase the risk of this disease.  2. Immune system issues: Crohn's disease may be linked to abnormal immune system responses. The immune system may mistakenly attack normal cells and microbes in the intestines, leading to inflammation and damage.  3. Environmental factors: Certain environmental factors, particularly smoking and dietary habits, may be related to the onset of Crohn's disease. Some studies suggest that smoking may increase the risk of developing Crohn's disease, while a high-fiber diet and intake of antioxidants may help reduce the risk.  4. Intestinal microbiota: The microbial community in the intestines (intestinal microbiota) may play a role in the onset of Crohn's disease. Imbalances in the intestinal microbiota may trigger inflammatory responses.  5. Lifestyle and psychological factors: Lifestyle factors such as stress, smoking, and substance abuse, as well as psychological factors, may be associated with the onset and severity of Crohn's disease. (expansion of content)  It is important to note that while these factors may increase the risk of developing Crohn's disease, they do not necessarily mean that everyone will develop the disease. Furthermore, research is ongoing, and our understanding of the causes and mechanisms of Crohn's disease is not yet complete. If you suspect you have Crohn's disease or have already been diagnosed, it is essential to consult healthcare professionals for expert advice and treatment plans. They can tailor the most appropriate treatment plan based on your specific circumstances. (open dialogue, professional guidance) | Crohn's disease is a chronic inflammatory bowel disease (IBD) with an unclear exact cause, but multiple factors may increase the risk of individuals developing this condition. Some potential factors include: (conceptual clarification)  1. Genetic factors: Individuals with a family history of Crohn's disease are more prone to the illness, suggesting that genetic factors may play a role.  2. Immune system issues: Crohn's disease is believed to be one of the results of an exaggerated immune system response to normal intestinal bacteria and food. Immune system abnormalities may make certain individuals more susceptible to this disease.  3. Environmental factors: Certain environmental factors such as diet, smoking, medications, and infections may be associated with the risk of developing Crohn's disease.  4. Intestinal microbiota: The composition of the intestinal microbiota may play a role in the development of Crohn's disease. Studies indicate that the interaction between intestinal microbiota and the immune system may influence the progression of Crohn's disease.  5. Other factors: Additional factors such as psychological stress, lifestyle choices, and dietary habits may also impact the risk of developing Crohn's disease. (expansion of content)  It is important to note that the specific causes of Crohn's disease vary among individuals and may result from the interaction of multiple factors. If you have been diagnosed with Crohn's disease, it is advisable to consult with a healthcare professional to gain more insights into your personal situation and develop the most suitable treatment plan and management approach. If you are concerned about the risk of developing Crohn's disease, it is also recommended to discuss potential preventive measures and early screening methods with your doctor. (open dialogue, professional guidance) |
| 克罗恩病是一种慢性炎症性肠病，其确切原因尚不清楚，但研究表明它可能是由多种因素相互作用引起的。虽然我们不能为您提供个人化的医疗建议，但以下是一些可能导致克罗恩病的因素和风险因素：（概念释义，开放话术）  1遗传因素：有家族史的人可能更容易患上克罗恩病。遗传因素可能在该疾病的发病中起到一定的作用。  2免疫系统问题：克罗恩病被认为是免疫系统异常反应的结果，免疫系统可能错误地攻击肠道内的正常细胞和细菌，导致炎症。  3环境因素：一些环境因素，如吸烟、饮食、感染和暴露于某些化学物质，可能与克罗恩病的发病有关。  4.肠道微生物：肠道内的微生物群可能对克罗恩病的发展起到一定作用。微生物的不平衡可能会引发炎症反应。  5.其他因素：精神压力、药物滥用、自身免疫性疾病等因素也可能增加患克罗恩病的风险。（内容展开）  请记住，克罗恩病的确切原因因人而异，每个人都可能受到不同的因素影响。如果您怀疑自己患有克罗恩病或有相关症状，建议咨询医生以获取专业的诊断和治疗建议。医生将根据您的病史、体格检查和可能的诊断测试来确定是否患有克罗恩病，并提供合适的治疗方案。（开放话术，专业引导） | 克罗恩病是一种慢性炎症性肠病（Inflammatory Bowel Disease，IBD），其确切原因尚不清楚，但可能与多种因素相互作用有关，包括遗传、免疫系统、环境和生活方式等。尽管研究仍在进行中，但以下因素可能与克罗恩病的发病有关：（概念释义）  1遗传因素： 有家族史的人患克罗恩病的风险较高。研究发现，某些基因变异可能增加患该病的风险。  2免疫系统问题： 克罗恩病可能与免疫系统的异常反应有关。免疫系统可能错误地攻击肠道内的正常细胞和微生物，导致炎症和损害。  3环境因素： 某些环境因素可能与克罗恩病的发病有关，尤其是吸烟和饮食习惯。一些研究表明，吸烟可能增加患克罗恩病的风险，而高纤维饮食和抗氧化物质摄入可能有助于降低患病风险。  4肠道微生物群： 肠道内的微生物群（肠道菌群）在克罗恩病的发病中可能起到一定作用。不正常的肠道菌群可能导致炎症反应。  5生活方式和心理因素： 压力、抽烟、药物滥用等生活方式和心理因素可能与克罗恩病的发病和病情严重程度有关。（内容展开）  需要注意的是，虽然这些因素可能增加患克罗恩病的风险，但并不一定导致每个人都会患上这种病。此外，研究仍在进行中，我们对克罗恩病的起因和发展机制尚不完全了解。如果您怀疑自己患有克罗恩病或已被确诊，请务必咨询医疗专家以获取专业建议和治疗方案。他们可以根据您的具体情况制定最合适的治疗计划。（开放话术，专业引导） | 克罗恩病是一种慢性炎症性肠道疾病（IBD），其确切原因尚不清楚，但有多种因素可能会增加患者患上这种病的风险。一些可能的因素包括：（概念释义）  1遗传因素：克罗恩病在家族中有发病史的人更容易患病，表明遗传因素可能在其中起到一定作用。  2免疫系统问题：克罗恩病被认为是免疫系统对正常肠道细菌和食物反应过度的结果之一。免疫系统异常可能使某些人更容易患上这种疾病。  3环境因素：某些环境因素，如饮食、吸烟、药物和感染，可能与克罗恩病的发病风险有关。  4.肠道微生物：肠道内的微生物组成可能对克罗恩病的发展起到一定作用。研究表明，肠道微生物与免疫系统的相互作用可能影响克罗恩病的发展。  5其他因素：一些其他因素，如精神压力、生活方式和饮食选择，也可能影响克罗恩病的发病风险。（内容展开）  需要注意的是，克罗恩病的具体原因因个体而异，可能是多种因素的相互作用结果。如果您已经被诊断出患有克罗恩病，最好咨询医生以了解更多关于您个人情况的信息，以制定最合适的治疗计划和管理方法。如果您担心患上克罗恩病的风险，也建议与医生讨论可能的预防措施和早期筛查方法。（开放话术，专业引导） |
